# Ash1l Methylates Lys36 of Histone H3 Independently of Transcriptional Elongation to Counteract Polycomb Silencing

**DOI:** 10.1371/journal.pgen.1003897

**Published:** 2013-11-07

**Authors:** Hitomi Miyazaki, Ken Higashimoto, Yukari Yada, Takaho A. Endo, Jafar Sharif, Toshiharu Komori, Masashi Matsuda, Yoko Koseki, Manabu Nakayama, Hidenobu Soejima, Hiroshi Handa, Haruhiko Koseki, Susumu Hirose, Kenichi Nishioka

**Affiliations:** 1 Division of Molecular Genetics and Epigenetics, Department of Biomolecular Sciences, Faculty of Medicine, Saga University, 5-1-1 Nabeshima, Saga City, Saga, Japan; 2 Precursory Research for Embryonic Science and Technology (PRESTO), Japan Science and Technology Agency (JST), 4-1-8 Honcho, Kawaguchi City, Saitama, Japan; 3 Division of Gene Expression, Department of Developmental Genetics, National Institute of Genetics, 1111 Yata, Mishima City, Shizuoka, Japan; 4 RIKEN Center for Integrative Medical Sciences, RIKEN Yokohama Institute, 1-7-22 Suehiro-cho, Tsurumi-ku, Yokohama City, Kanagawa, Japan; 5 Graduate School of Bioscience and Biotechnology, Tokyo Institute of Technology, 4259 Nagatsuta, Yokohama City, Kanagawa, Japan; 6 Laboratory of Medical Genomics, Department of Human Genome Research, Kazusa DNA Research Institute, 2-6-7 Kazusa-kamatari, Kisarazu City, Chiba, Japan; 7 Core Research for Evolutional Science and Technology (CREST), Japan Science and Technology Agency (JST), 4-1-8 Honcho, Kawaguchi City, Saitama, Japan; Centre National de la Recherche Scientifique, France

## Abstract

Molecular mechanisms for the establishment of transcriptional memory are poorly understood. 5,6-dichloro-1-D-ribofuranosyl-benzimidazole (DRB) is a P-TEFb kinase inhibitor that artificially induces the poised RNA polymerase II (RNAPII), thereby manifesting intermediate steps for the establishment of transcriptional activation. Here, using genetics and DRB, we show that mammalian Absent, small, or homeotic discs 1-like (Ash1l), a member of the trithorax group proteins, methylates Lys36 of histone H3 to promote the establishment of Hox gene expression by counteracting Polycomb silencing. Importantly, we found that Ash1l-dependent Lys36 di-, tri-methylation of histone H3 in a coding region and exclusion of Polycomb group proteins occur independently of transcriptional elongation in embryonic stem (ES) cells, although both were previously thought to be consequences of transcription. Genome-wide analyses of histone H3 Lys36 methylation under DRB treatment have suggested that binding of the retinoic acid receptor (RAR) to a certain genomic region promotes trimethylation in the RAR-associated gene independent of its ongoing transcription. Moreover, DRB treatment unveils a parallel response between Lys36 methylation of histone H3 and occupancy of either Tip60 or Mof in a region-dependent manner. We also found that Brg1 is another key player involved in the response. Our results uncover a novel regulatory cascade orchestrated by Ash1l with RAR and provide insights into mechanisms underlying the establishment of the transcriptional activation that counteracts Polycomb silencing.

## Author Summary

Transcriptional mechanisms in eukaryotes are composed of numerous consecutive steps, including chromatin modification and remodeling. Recent reports using yeast genetics have revealed that Lys36 methylation of histone H3, a hallmark of the active gene, is a consequence of transcriptional elongation. Similarly, a report using *Drosophila* genetics showed that exclusion of the Polycomb repressive complexes, general repressor complexes that regulate development and cellular differentiation, is another consequence of transcription. Here, we provide evidence that these causal relationships are not really general. By ceasing ongoing transcription at a certain step using an inhibitor in combination with mouse genetics, we have identified novel intermediate steps of transcription: Ash1l-mediated Lys36 methylation of histone H3 and subsequent exclusion of the Polycomb complexes that occur independently of transcriptional elongation. Furthermore, we show that binding of a nuclear receptor may promote trimethylation of Lys36 in its associated gene independent of its ongoing transcription. In this paper, we detail previously unknown key machineries orchestrated against Polycomb silencing, providing an innovative view of the mechanisms involved in the establishment of transcriptional memory.

## Introduction

Studies on the regulation of transcriptional memory are challenging. Conceptually, the regulation consists of two phases: establishment and maintenance. Molecular mechanisms for the maintenance of the memory are relatively well understood compared with those for the establishment of memory. Indeed, how the establishment of transcriptional activation occurs is largely unknown because it has been difficult to distinguish mechanisms for establishment from those for maintenance, presumably due to functional redundancies and spatial and temporal overlap between them. Moreover, if transient regulation is involved during the establishment phase, it is extremely difficult to tease apart and analyze the respective underlying mechanisms.

For the establishment of transcriptional activation of developmentally regulated genes in stem cells, we know that the poised RNAPII should be released from pausing in the promoter-proximal coding region, as occurs in response to various microenvironmental cues [1], and that the associated chromatin should be kept competent for transcription by RNAPII throughout a coding region. P-TEFb, a cyclin-dependent kinase complex, plays a pivotal role in the RNAPII pause release by alleviating the repressive effects of DRB sensitivity-inducing factor (DSIF) and negative elongation factor (NELF), and by phosphorylating the Ser2 residue of the carboxyl-terminal domain of RNAPII [2]–[5]. In addition to recruitment of P-TEFb, it has been proposed that recruitment of a certain chromatin remodeling factor is also crucial to the release of paused RNAPII [3], which appears to be situated adjacent to the first nucleosome downstream of the transcription start site [6]. Thus, it seems that at least two independent mechanisms are required to trigger productive transcriptional elongation. It is conceivable that these mechanisms are engaged throughout the coding region to maintain active gene expression. In addition, the activation of Polycomb group-target genes further requires several counteracting mechanisms against the Polycomb repressive complexes (PRCs) [7]. However, these mechanisms underlying the establishment of transcriptional memory and how these mechanisms are orchestrated remain elusive.

Ash1l is the mammalian equivalent of the *Drosophila* Ash1 protein. Although Ash1 is one of the first identified members of the trithorax group proteins [7], both the Ash1- and Ash1l-containing complexes remain uncharacterized. Both Ash1 and Ash1l are localized in chromatin and have been identified specifically in promoter-proximal coding regions of a number of active genes [8], [9], suggesting a role during an early step of transcriptional elongation. Additionally, artificial tethering of Ash1 to chromatin containing a reporter gene results in gene activation in a SET domain-dependent manner [10]. These results suggest that Ash1 is an epigenetic activator found in an “ON” state of target genes, although the underlying mechanism of its action remains unknown.

Like a number of SET domain-containing proteins, both Ash1 and Ash1l possess histone lysine methyltransferase activity. However, it is currently controversial as to which lysine residue is targeted *in vivo*, although recent reports have suggested that Lys4 and Lys36 of histone H3 are the most plausible candidates [8]–[11]. So far, Lys4 is widely believed as a target residue due to several lines of *in vivo* evidence [8]–[10], compared with only one for Lys36 [12]. It is consistent with the activator function of Ash1 [10], while Lys36 methylation (Lys36me) has been shown to occur as a consequence of transcription [13]–[15]. However, it should be noted that an analysis of the enzymatic activities of Ash1 and Ash1l *in vivo* has been difficult due to their physical and functional interactions with other enzymes that methylate Lys4 [16], [17] and also due to the redundancy of enzymes methylating Lys36, such as Setd2, Nsd1, Nsd2 and Nsd3 in mammals. Moreover, expression of the full-length recombinant Ash1l and detection of the endogenous Ash1l by immunoblot has been as yet unsuccessful, thereby making analyses more challenging.

To elucidate the molecular mechanism of the Ash1l-mediated establishment of gene expression, we developed a knock-in allele expressing mutant of Ash1l without part of the SET domain. Using the mutant ES cells, we show that Ash1l methylates Lys36 of histone H3 both *in vitro* and *in vivo*. Importantly, using DRB, a P-TEFb kinase inhibitor that blocks productive transcriptional elongation, we show that the methylation by Ash1l and its effect on PRC exclusion occur independently of productive transcriptional elongation. In particular, an accumulation of Lys36me3 in RAR-associated genes independent of transcriptional elongation implicates a certain special function for establishing transcriptional activation of Polycomb group-target genes. Moreover, the broad chromatin domains carrying Lys36me were co-regulated with the Tip60 or Mof complexes in a region-dependent manner, which in turn acetylate the Lys16 of histone H4. We further investigated a mechanism for the promotion of gene expression by Ash1l, and found an Ash1l-dependent association with Brg1, another founding member of the trithorax group proteins. These molecular data in ES cells are supported by expression patterns of Hox genes and skeletal phenotypes in Ash1l mutant mice. Thus, through genetic and biochemical analyses of Ash1l, we have elucidated a novel cascade of interplays from Ash1l to Brg1, which ultimately promotes chromatin reprogramming that counteracts Polycomb silencing.

## Results

### *Ash1l* knock-in mutant ES cells demonstrate impaired retinoic acid response

**Figure 1:**
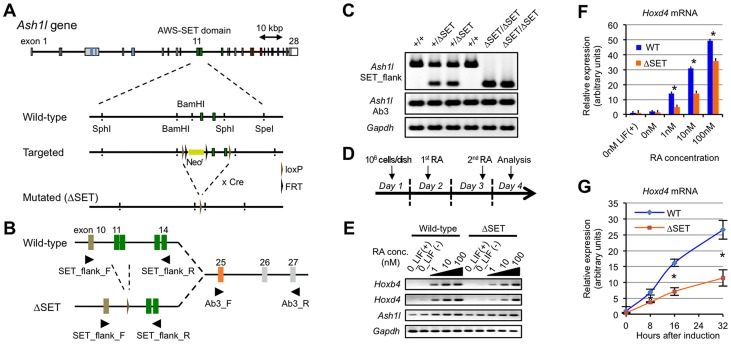
Basic characterization of Ash1l mutant ES cells and Hox gene expression in response to RA. (**A**) Schematic representation of the strategy used for targeted disruption of the *Ash1l* gene. Exons 11–12 encoding the AWS-SET domain with their flanking introns were floxed by loxP sequences. Cre-mediated germ-line recombination resulted in the generation of the ΔSET allele. (**B**) A PCR primer-pair of SET_flank_F/R was used to distinguish between expression from the wild-type allele and that from the ΔSET allele as shown in (A). A PCR primer-pair of Ab3_F/R was used to determine total expression from both alleles. The PCR primer-pairs are listed with their sequences in Table S4. (**C**) Comparison of *Ash1l* mRNA expression among E10.5-litter-mate embryos by conventional RT-PCR. Expression levels of *Gapdh* mRNA are shown as controls. (**D**) Protocol for RA-induced differentiation of ES cells. (**E**) Conventional RT-PCR analyses of *Hoxb4* and *Hoxd4* mRNA expression levels in response to various concentrations of RA. (**F** and **G**) Quantitative RT-PCR analysis of *Hoxd4* mRNA expression levels. RA-titration analysis after 48 hours of induction (F), and a time-course analysis using 1 nM RA (G). The results are represented as relative expression levels between wild-type and ΔSET ES cells. Wild-type cells (blue bars or line) and homozygous ΔSET ES cells (orange bars or line) are shown. These results represent means and standard deviations (s.d.) of three independent PCR reactions (Student's t-test, *P<0.05).

To explore a function of the methyltransferase activity of mammalian Ash1l, we generated knock-in mice and ES cells expressing mutant Ash1l containing a short in-frame deletion within the AWS-SET domain (represented by ΔSET, Figure 1A). Mouse *Ash1l* mRNA was ubiquitously expressed in embryos, while it was relatively enriched in the adult brain (Figures S1A, S1B and data not shown). Expression of *Ash1l* mRNA was not affected in ΔSET embryos and ES cells (Figures 1B and 1C). Homozygotes were viable and fertile (Table S1).

Since Ash1l is a member of the trithorax group proteins that regulate transcriptional activation of Hox genes, and retinoic acid (RA) is known to induce expression of Hox genes in ES cells, a role for Ash1l methyltransferase activity in RA-induced Hox gene expression was investigated in differentiating ES cells. A culture protocol is shown in Figure 1D. Here, we analyzed representative RA-responsive Hox genes, *Hoxb4*, and *Hoxd4*. Using a series of RA concentrations, we found that expression levels of *Hoxb4* and *Hoxd4* mRNAs were reduced in ΔSET ES cells (Figure 1E). Specifically, the threshold concentration of RA required to trigger the *Hoxd4* mRNA expression was significantly increased in ΔSET ES cells compared with that in wild-type cells (Figure 1F). Moreover, the activation of the *Hoxd4* mRNA expression was relatively slow in ΔSET ES cells (Figure 1G). These results indicate that the methyltransferase activity of Ash1l is necessary for an appropriate response to RA.

**Figure 2:**
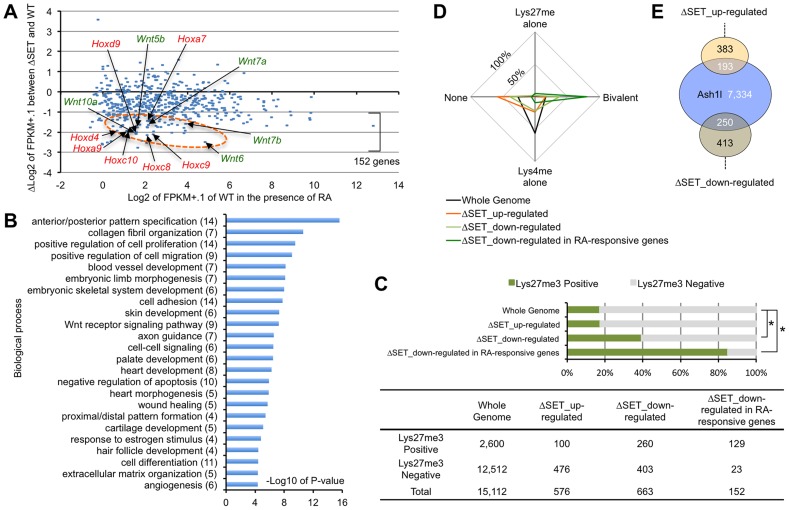
Comprehensive gene expression analyses of differentiating ES cells. ES cells were treated with 10-Seq analyses. (**A**) RA-responsive genes (those whose expression levels were increased more than 5-fold compared with undifferentiated ES cells: 543 genes out of 14,255 eligible annotated genes) were plotted on the graph using modified fragments/kb of transcript/million fragments mapped (FPKM) values. The x-axis corresponds to expression levels of each gene (shown as log2 transformation of each FPKM value plus 0.1), and the y-axis corresponds to fold change in gene expression levels between ΔSET ES cells and wild-type (shown as Δlog2 transformation). The encircled area was enriched for some Hox and Wnt family genes (arrows, see also Figure S2). (**B**) Gene ontology analysis of ΔSET-impaired 152 genes. A subset of RA-responsive genes demonstrating a greater-than-2-fold decrease in the modified FPKM values in differentiating ΔSET ES cells was analyzed. Gene enrichment P-values were calculated by Chi-square test. The numbers of genes in each group are shown in parentheses. (**C**) Bar chart showing relative ratios of the numbers of genes carrying trimethylation of Lys27 [18], a hallmark of the Polycomb-regulated genes. Genes showing a difference greater than 2-fold were analyzed (up- or down-regulated). In addition, RA-responsive genes in the down-regulated genes were further extracted and analyzed [the last group, same as in (B)]. Chi-square testing was used for calculation of P-values against the total gene set, *P<0.001. The numbers of genes in each group are shown in the table below. (**D**) Radar chart showing relative ratio of status of chromatin signatures for indicated gene group as in (C). (**E**) Venn diagram showing the relationship between Ash1l-target genes and ΔSET-affected genes. The numbers of genes in each compartment are shown. The total number of annotated genes analyzed was 18,724.

We further performed gene expression analyses of RA-treated differentiating ES cells by RNA-sequencing (RNA-Seq) to determine if RA-responsive genes were generally affected in ΔSET ES cells. Among 14,255 annotated genes, there were 543 genes that were highly up-regulated by RA treatment (more than 5-fold). Among those 543 genes, we found 152 genes (28.0%) in ΔSET ES cells showing impaired responses to RA (more than 2-fold decrease compared with wild-type cells, Figure 2A and Table S2), in which several Wnt and Hox family genes appeared to be affected (Figure S2). As expected, gene ontology analysis of the 152 genes revealed that biological functions of Ash1l were highly related to body pattern formation during development (Figure 2B).

The status of chromatin signatures in ES cells can be classified in terms of the presence of Lys4me3 or Lys27me3 in histone H3 polypeptides: Lys4me alone, Bivalent (positive for both Lys4me3 and Lys27me3), Lys27me alone and None (negative for both Lys4me3 and Lys27me3) [18]. Interestingly, the 152 RA-responsive and ΔSET-impaired genes were significantly enriched in a group positive for Lys27me3 (Figure 2C) and further enriched especially in the Bivalent gene group (Figure 2D), suggesting that the methyltransferase activity of Ash1l counteracts Polycomb silencing upon activation of developmentally regulated genes. Chromatin immunoprecipitation-sequencing (ChIP-Seq) analysis revealed that Ash1l was present in more than 30% of those affected genes (Figure 2E).

RNA-Seq analysis of undifferentiated ES cells showed that expression levels of a majority of marker genes, including core stem cell markers, were unchanged, while some endoderm markers were moderately up-regulated in ΔSET ES cells (Figure S3A). RA treatment of ES cells further enhanced the up-regulation (Figure S3B). Comprehensive analysis of RNA expression levels of 14,255 annotated genes revealed that 57 genes were down-regulated more than 2-fold in undifferentiated ΔSET ES cells, while 59 genes were up-regulated (Table S3). Several microRNAs and *Snord* family genes were highly dys-regulated in ΔSET ES cells, although the impact of the methyltransferase activity of Ash1l on these genes *in vivo* remains unclear. While this manuscript was under preparation, a report describing the methyltransferase activity of Ash1l for Hox gene repression was published online [19]. Reason for discrepancy between our results and theirs is currently unknown. However, conditions of basic materials and methods may affect each result: knock-in mutant ES cells and mice for ours, exogenous transient over-expression in K562 cells for theirs.

### Ash1l methylates Lys36 of histone H3 *in vitro*

**Figure 3:**
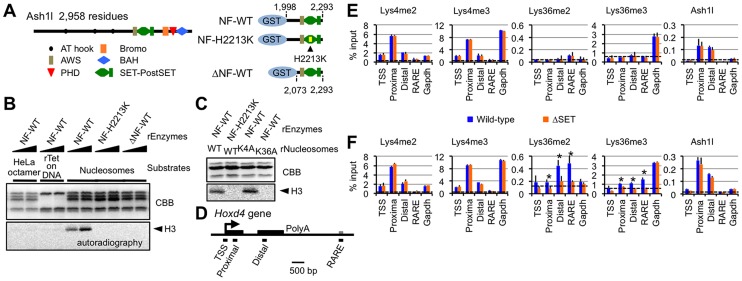
Ash1l specifically methylates Lys36 of histone H3 both *in vitro* and *in vivo*. (**A**) Structure of full-length Ash1l (left) and GST-fusion Ash1l constructs (right). NF: the N-terminal flanking region of the AWS domain, H2213K: amino acid substitution of His2213 to Lys. (**B** and **C**) Histone methyltransferase assays with GST-fusion Ash1l (rEnzyme). rTet on DNA: recombinant (H3–H4)_2_ tetramers bound to plasmid DNA. rNucleosomes: recombinant nucleosomes containing wild-type or mutant histone H3 as indicated. CBB: Coomassie brilliant blue staining for histone polypeptides separated on a SDS-PAGE gel. Position of histone H3 in the autoradiogram is indicated in each assay (arrowheads). (**D**) Diagram of the *Hoxd4* gene. Black and grey boxes represent exons and a 3′ retinoic acid responsive element (RARE), respectively. Black bars under the diagram indicate the regions analyzed by ChIP assays. TSS: transcription start site. (**E** and **F**) ChIP assays of various regions in differentiating ES cells before (E) or after (F) RA treatment (see the culture protocol shown in Figure 1D). The results are represented as mean values relative to input (% input). Error bars represent s.d. (Student's t-test, *P<0.05). The antibodies used are indicated above each graph. Broken lines indicate approximate levels of ChIP signals in the *Il2ra* promoter as a control.

To elucidate an underlying mechanism involved in the regulation of gene expression by Ash1l, we examined the biochemical activity of Ash1l *in vitro*. We employed a bacterially expressed GST-fusion Ash1l protein (Figure 3A) since it has no associated protein that methylates histone polypeptides. As shown in Figure 3B, wild-type Ash1l (NF-WT) methylated histone H3 in nucleosomes, but did not methylate free histone octamers or (H3-H4)_2_ tetramers bound to DNA. Addition of H2A-H2B dimers to the (H3-H4)_2_ tetramers on DNA in the reaction mixture failed to replicate the methylation activity [K.N. unpublished observation], and an amino acid substitution within the core catalytic part of the SET domain (NF-H2213K) abolished it. Additionally, wild-type Ash1l methylated wild-type histone H3 and its Lys4-to-Ala (K4A) mutant to a similar extent, but not its Lys36-to-Ala (K36A) mutant (Figure 3C). These results clearly indicate that Ash1l specifically methylates Lys36 of histone H3 and presumably recognizes the preinstalled H2A-H2B dimer in a nucleosome to target Lys36. Furthermore, recombinant Ash1l carrying a deletion at the N-terminal flanking region of the AWS domain (ΔNF-WT) was catalytically inactive, indicating that this region is necessary for optimum methyltransferase activity.

### Ash1l methylates Lys36 of histone H3 upon gene activation

We next examined whether our *in vitro* results could be recapitulated in an *in vivo* setting. Histone modification patterns and Ash1l occupancy in *Hoxd4* in differentiating ES cells were analyzed by ChIP assays. PCR primer-pairs were set as shown in Figure 3D (Table S4 for sequences). A promoter-proximal coding region of *Gapdh* was also investigated as a constitutively active control. *Hoxd4* chromatin has been reported to be bivalent in undifferentiated ES cells [18]. Consistent with the report, even in the absence of RA, peaks of histone H3 Lys4me2 and me3 (Lys4me2/3) were clearly detected in a promoter-proximal coding region of *Hoxd4*, while the levels of histone H3 Lys36me2 and me3 (Lys36me2/3) were rudimentary (Figure 3E). Surprisingly, Ash1l was clearly present in *Hoxd4* even in the absence of RA (Figure 3E). We also observed considerable amounts of Ash1l in the promoter-proximal coding region of *Gapdh*. However, we observed no difference between wild-type and ΔSET ES cells in these ChIP assays.

Following RA treatment, while the levels of Lys36me2/3 were increased in *Hoxd4* coding regions in wild-type cells, this was not observed in ΔSET ES cells (Figure 3F). There was no significant change in the levels of Lys4me2/3 of *Hoxd4* in ΔSET ES cells. We found that the levels of Ash1l in coding regions of *Hoxd4* and *Gapdh* were maintained after RA treatment. We observed no difference in the levels of Ash1l between wild-type and ΔSET ES cells. These results indicate that Ash1l specifically methylates Lys36 *in vivo*, as expected from our *in vitro* results, and suggest that the enzymatic activity of Ash1l is activated upon the addition of RA. Since Ash1l is a dimethylase [11], the observation that there was no increased Lys36me3 in ΔSET ES cells may be a consequence of an impaired Lys36me2-platform that is required as a substrate for a certain trimethylase.

### Lys36me occurs independently of RNAPII Ser2p

Based on several studies conducted in yeast, the presence of Lys36me in a coding region is widely believed to be a consequence of transcriptional elongation [13]–[15] and to function to recruit the histone deacetylase complex [20], [21]. However, our results suggested a novel hypothesis: Lys36me by Ash1l in a coding region occurs during the establishment of Hox gene activation to promote a proper response to RA (see Figures 1F and 1G). Therefore, in this case, the Lys36me should be independent of the productive transcriptional elongation.

To test our hypothesis, we employed DRB, which reversibly blocks productive transcriptional elongation by inhibiting the kinase activity of P-TEFb. DRB artificially creates the poised RNAPII, closely mimicking the promoter-proximally paused RNAPII in a gene demonstrating bivalent chromatin in ES cells, thereby manifesting intermediate steps for the establishment of transcriptional activation. If Lys36me by Ash1l is independent of the RNAPII Ser2p or transcriptional elongation, then DRB would not affect methylation levels. In the next experiments, DRB was added to differentiating ES cells during RA treatment and ChIP assays were performed. Two Ash1l-associated genes were compared: *Hoxd4*, representing a Polycomb group-target gene, and *Gapdh*, a constitutively active gene.

**Figure 4:**
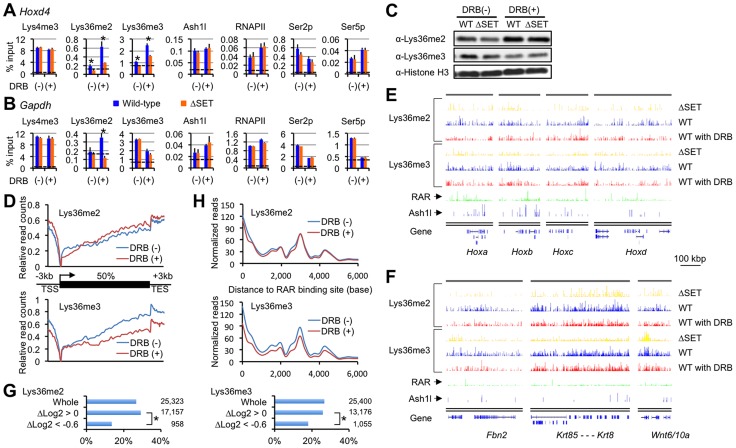
Lys36me2 occurs independently of the Ser2-phosphorylation of RNAPII, while Lys36me3 occurs in a context-dependent manner. ChIP assays of differentiating ES cells either with (+) or without (−) DRB treatment. Promoter-proximal coding regions of either *Hoxd4* (**A**) or *Gapdh* (**B**) were analyzed. DRB was added during RA treatment, and then ES cells were cultured for another 16 hours. The antibodies used are indicated above each graph. The results are represented as means and s.d. (Student's t-test, *P<0.05). Broken lines indicate approximate levels of ChIP signals in either *Il2ra* promoter as controls. (**C**) In the presence or absence of DRB, bulk histones in differentiating ES cells were analyzed by immunoblot using the indicated antibodies. (**D**) Distributions of Lys36me2/3 ChIP-Seq read counts relative to a metagene in the presence or absence of DRB. The y-axis corresponds to relative read counts to base-read counts (shown as log2 transformation). TSS: transcription start site, TES: transcription end site. (**E** and **F**) Genomic profiles for Lys36me2/3, RAR, and Ash1l ChIP-Seq signals of numbers of genomic loci in differentiating ES cells (10 nM RA treatment for 2 days). DRB was used during culturing of cells where indicated. The x-axis corresponds to genomic locations; the y-axis corresponds to the normalized ChIP-Seq signal density. Representations of UCSC genes are shown on the bottom, in which Hox loci (E) and the other representative loci (F) are shown. RAR ChIP-Seq datasets (GSE19409) [22] were downloaded from the NCBI Short Read Archive database. (**G**) Bar charts showing relative ratios of the numbers of RAR-associated genes in indicated gene groups in differentiating ES cells. The gene groups are classified according to fold change in Lys36me2/3 levels in response to DRB: Δlog2 transformation of normalized reads/kb/million mapped (RPKM) values of each gene in DRB (+) over those in (−). Total numbers of genes in each gene group are indicated on the right side of each graph. Chi-square testing was used for calculation of P-values where indicated, *P<0.001. (**H**) Distributions of Lys36me2/3 ChIP-Seq read counts relative to RAR binding sites in ES cells in the presence or absence of DRB.

As shown in Figure 4A, far from a decrease in the Lys36me level, wild-type ES cells displayed a clear increase in Lys36me2/3 levels in the promoter-proximal coding region of *Hoxd4* in response to DRB. Interestingly, differences in the Lys36me2/3 levels between wild-type and ΔSET ES cells were more evident in the presence of DRB. On the other hand, DRB also increased the Lys36me2/3 levels in ΔSET ES cells, albeit to a lesser extent [compare (−) and (+) in ΔSET], suggesting that some Lys36-methylases other than Ash1l could be involved, although the identity of the enzyme recruited to the region has yet to be determined. In *Gapdh*, DRB increased Lys36me2 levels in wild-type cells, but DRB treatment resulted in a clear decrease in Lys36me3 levels (Figure 4B), suggesting that DRB specifically affected association of a certain trimethylase that was recruited in a RNAPII Ser2p-dependent manner. Similar results were obtained for *Hoxb4* and *Hprt1* (Figure S4). These results indicate that Lys36me2 by Ash1l and other dimethylases occurs independently of RNAPII Ser2p. However, whether Lys36me3 occurs independently of RNAPII Ser2p is gene-dependent.

### Lys36me2 in a large number of regions occurs independently of the Ser2-phosphorylation of RNAPII, while Lys36me3 occurs in a context-dependent manner

Having established that *Hoxd4* and *Gapdh* were DRB-responsive genes in that Lys36me2(/3) was increased in response to DRB, we analyzed if the observed response was applicable genome-wide. First, immunoblot analyses for bulk Lys36me2 and Lys36me3 levels were performed (Figure 4C). In the presence of RA, we found the bulk Lys36me2 levels in wild-type and ΔSET ES cells were similarly increased in response to DRB, while those of Lys36me3 were not, and instead decreased.

Next, we performed ChIP-Seq analyses to obtain genome-wide profiles of Lys36me2/3 in response to DRB. Distributions of Lys36me2/3 relative to a metagene show a clear difference between Lys36me2 and me3 in response to DRB: Lys36me3 levels were decreased in isolated, entire regions, while in contrast, Lys36me2 levels were increased in most of the regions (Figure 4D). Thus, having observed that Lys36me3 of *Hoxd4* was increased in response to DRB, here we found that its genome-wide level showed the opposite response, indicating that genes demonstrating increased Lys36me3 levels in response to DRB were minorities.

Figures 4E and 4F show profiles of the representative genomic regions. In these genomic profiles, we found that the level of Lys36me2 in wild-type cells was generally unchanged in response to DRB in a broad range of regions, or rather increased in some parts, while that of Lys36me3 was decreased in most regions. However, we also found that substantial levels of Lys36me3 remained in scattered regions, including some inter-genic regions. Interestingly, quite a few such regions were found in the vicinity of RAR binding sites [22], implicating a functional relationship between Lys36me3 in response to DRB and RA signaling. Indeed, the numbers of RAR-associated genes were significantly underrepresented in a gene group with decreased levels of Lys36me3 in response to DRB (Figure 4G, ΔLog2<−0.6). Most strikingly, RAR binding sites showed a major peak in the Lys36me3 ChIP-Seq read density plot (Figure 4H). Additionally, in B16 cells, which express *Hoxd4* constitutively without the addition of RA, we observed only a small increase in Lys36me3 levels in response to DRB (Figure S5). These results further support our proposed relationship. Taken together, our results indicate that Lys36me2 by Ash1l and other dimethylases occurs independently of RNAPII Ser2p in a large number of genomic regions. However, whether Lys36me3 occurs independently of RNAPII Ser2p is context-dependent, and at least in a portion of genomic regions, RAR may play a substantial role for maintaining Lys36me2/3 levels.

### Lys36me occurs independently of productive transcriptional elongation

**Figure 5:**
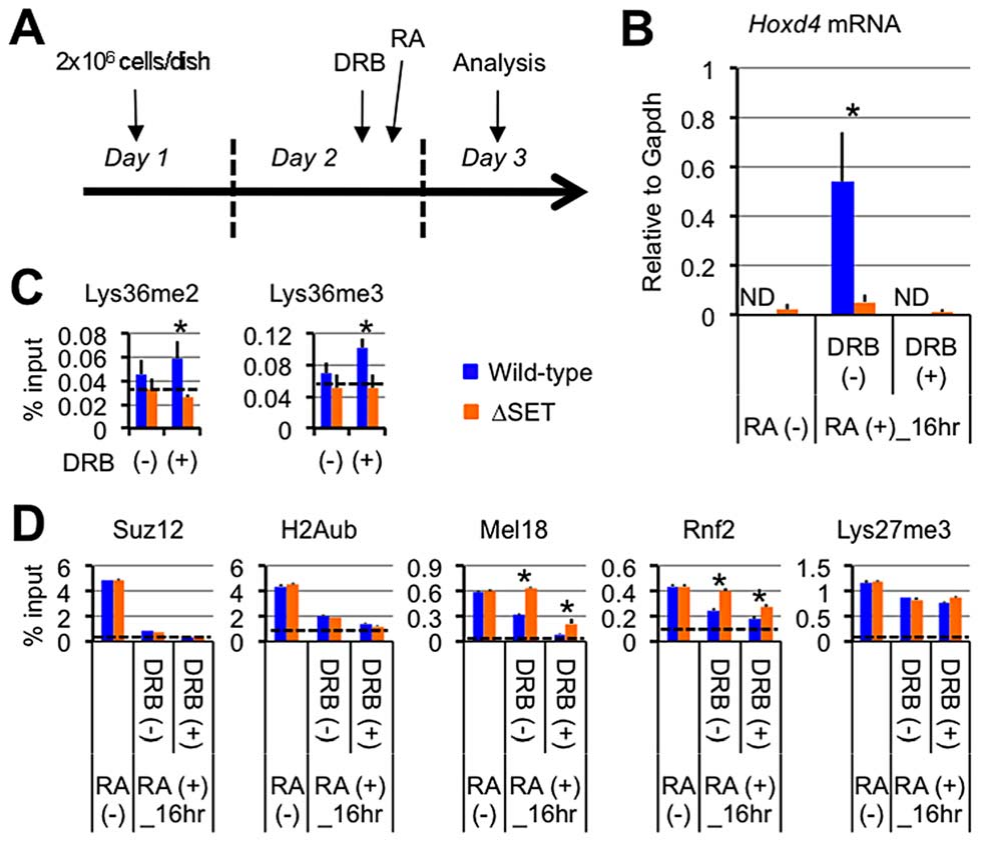
Lys36me2/3 and exclusion of the PRCs occur independently of productive transcriptional elongation. (**A**) Protocol for RA-induced differentiation of ES cells under DRB pretreatment. DRB was added to the culture medium 1 hour before the addition of RA, then the ES cells were cultured for another 16 hours in the presence of 100 nM RA. (**B**) Nuclear run-on assay in combination with RT-qPCR analyses of *Hoxd4* mRNA expression either with or without DRB pretreatment. The results are represented as values relative to *Gapdh* mRNA in each cell type. Error bars represent s.d. (Student's t-test, *P<0.05). ND: not detected. (**C** and **D**) ChIP assays of differentiating ES cells either with (+) or without (−) DRB pretreatment. A promoter-proximal coding region of *Hoxd4* was analyzed. The antibodies used are indicated above each graph. The results are represented as means and s.d. (Student's t-test, *P<0.05). Broken lines indicate approximate levels of ChIP signals in either *Il2ra* promoter (C) or *Gapdh* coding region (D) as controls.

In the above experiments, it is possible that past productive transcriptional elongation had left unknown traces on the transcribed chromatin, which in turn was targeted by several Lys36-methylases including Ash1l, although recruitment of the methylases is independent of RNAPII Ser2p. Moreover, it remains unclear whether the activation of the enzymatic activity of Ash1l requires the productive transcriptional elongation. Therefore, in the next experiments, to block the productive transcriptional elongation completely, we used DRB prior to administering RA. Specifically, DRB was added 1 hour before the addition of RA, and then the cells were cultured for another 16 hours in the presence of RA (Figure 5A).

Under these conditions, *Hoxd4* mRNA was not increased at all from the basal level that was observed in undifferentiated ES cells, indicating that DRB blocked the productive transcriptional elongation completely (Figure 5B). As shown in Figure 5C, while wild-type ES cells displayed mild increases in Lys36me2/3 levels in the promoter-proximal coding region of *Hoxd4* in response to DRB, ΔSET ES cells did not, resulting in clear differences between wild-type and ΔSET ES cells in the presence of DRB. The results indicate that Ash1l-dependent Lys36me2/3 in *Hoxd4* occurs independently of productive transcriptional elongation during the establishment of transcriptional activation. This may be reasonable since Ash1l is preloaded on the *Hoxd4* chromatin before the addition of RA (see Figure 3E).

### Ash1l promotes exclusion of the PRC1 in a transcription-independent manner

Can transcription-independent Lys36me by Ash1l counteract Polycomb silencing? A previous report showed that transcription is necessary to exclude the PRCs from local chromatin [23]. However, proof remains elusive of whether progression of RNAPII itself is the major determinant factor for the exclusion. Moreover, how the PRCs are removed upon gene induction is poorly understood. Therefore, under the same conditions, i.e. the addition of DRB prior to RA, we characterized the status of Polycomb silencing in ES cells by analyzing Suz12 (a component of the PRC2), Lys27me3 (an enzymatic product of the PRC2), ubiquitination of histone H2A (H2Aub, an enzymatic product of the PRC1), Mel18, and Rnf2 (components of the PRC1) in *Hoxd4* chromatin.

We found significantly higher levels of Mel18 and Rnf2 in *Hoxd4* chromatin of ΔSET ES cells compared to wild-type cells in the absence and presence of DRB (Figure 5D). Interestingly, wild-type and ΔSET ES cells displayed clear decreases in Mel18 and Rnf2 levels upon blocking of transcription by DRB, demonstrating anti-parallel ChIP patterns against those of Lys36me2/3 (compare Figures 4A, 5C and 5D). Similar results were obtained for a distal coding region of *Hoxd4* (Figure S6C). Suz12 and H2Aub levels showed more rapid and clear decreases in response to RA. However, differences between wild-type and ΔSET ES cells in the occupancies of Suz12 and H2Aub were unclear, suggesting that there was an Ash1l-independent pathway to exclude these molecules. Lys27me3 levels displayed only a marginal response to both DRB and RA under these conditions (Figure 5D). However, we observed a clear decrease in Lys27me3 levels after a longer induction by RA, in which there was a substantial difference between wild-type and ΔSET ES cells (Figure S6D).

In summary, these experiments showed that Suz12, H2Aub, Mel18 and Rnf2 demonstrated relatively rapid responses to RA compared with Lys27me3, and contradicting a previous notion, their exclusion was not dependent on transcriptional elongation. Importantly, we found that exclusion of Mel18 and Rnf2 from chromatin upon RA induction was specifically impaired by loss of the methyltransferase activity of Ash1l, suggesting a negative relationship between PRC1 chromatin association and Ash1l activity. Although ΔSET ES cells displayed mild decreases in RNAPII Ser2-phosphorylation (Ser2p) levels in the coding regions of *Hoxd4*, the decreased levels of Lys36me did not affect the basic status of RNAPII for the most part (Figures S7A–S7C). Similar results were obtained even for a relatively larger gene, *Wnt6* (Figures S7D and S7E). These results suggest that the methyltransferase activity of Ash1l mainly contributes to promoting the anti-Polycomb silencing function rather than the activation of RNAPII directly.

### The functional link between Lys36me and Lys16ac in an entire coding region

Having established that Lys36me in *Hoxd4* occurs independently of productive transcriptional elongation and that DRB enhances the difference between wild-type and ΔSET ES cells, we next asked how the Lys36me facilitates transcriptional elongation. Given that certain histone acetylations have more direct effects on activation of transcription, ChIP assays were performed to analyze the effects of Lys36me on representative histone acetylations, such as Lys9/14 acetylation of H3 (Lys9/14ac) and Lys16 acetylation of H4 (Lys16ac). In all subsequent experiments, when necessary, DRB was added during RA treatment as in Figure 4.

**Figure 6:**
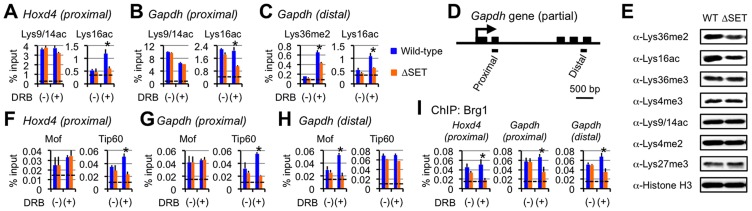
Functional links of Ash1l to the Tip60, Mof, and Brg1 complexes. (**A**–**C** and **F**–**I**) ChIP assays of *Hoxd4* and *Gapdh* in differentiating ES cells either with (+) or without (−) DRB treatment. Regions that were analyzed were divided into two parts as indicated in each panel: promoter-proximal (*proximal*) and distal (*distal*) coding regions. The antibodies used are indicated above each graph or in panels. The results are represented as means and s.d. (Student's t-test, *P<0.05). Broken lines indicate approximate levels of ChIP signals in *Il2ra* promoter as a control. (**D**) A diagram of the *Gapdh* gene is shown. Black bars under the diagram indicate the regions analyzed by ChIP assays. (**E**) Whole-cell extracts were analyzed by immunoblot using the antibodies against the indicated histone modifications.

Interestingly, the ChIP pattern of Lys16ac in *Hoxd4* was similar to that of Lys36me in that the ChIP signals were not decreased in the presence of DRB. In fact, they were increased in wild-type ES cells, and became clearly lower in ΔSET ES cells compared with wild-type cells (Figure 6A), thereby revealing the effect of Lys36me by DRB. The ChIP pattern of Lys9/14ac did not resemble even slightly that of Lys36me. These results collectively indicate that Lys16ac specifically correlates with Ash1l-dependent Lys36me, both of which are independent of RNAPII Ser2p. This was consistent with a recent report conducted in *Drosophila*, where connections were made between Lys36me2 with dMes-4 and Lys16ac by an unknown enzyme in proximal coding regions [24]. Interestingly, in our study, similar results were also obtained for *Gapdh* (Figure 6B), even in a further downstream distal coding region (Figures 6C and 6D), suggesting that cooperative action between Lys36-methylases including Ash1l and a certain Lys16 acetyltransferase influences these histone modifications in an entire coding region independently of RNAPII Ser2p. Similar results were obtained for *Hoxb4* and *Hprt1* (Figure S8), suggesting that the observed parallel link is a general phenomenon. In ΔSET ES cells, the global levels of Lys36me2 and Lys16ac, but not of Lys36me3, were found to be reduced (Figure 6E), which further corroborated the ChIP results.

### The Tip60 and Mof complexes are co-regulated with Lys36me in a region-dependent manner

Since we observed no significant difference in the levels of Ash1l, RNAPII, and Ser5p between wild-type and ΔSET ES cells (see Figure 4A), we speculated that Lys36me contributed to the association of a certain Lys16-acetyltransferase with a coding region chromatin. We next analyzed the Mof and Tip60 complexes as these complexes preferentially acetylate Lys16 of histone H4 and are highly relevant to transcriptional activation. Furthermore, since these complexes contain chromodomain proteins (Msl3l [25] in the Mof complex and Mrg15 [26] in the Tip60 complex) that bind Lys36-methylated histone H3 [27], [28], both complexes can associate with the Lys36-methylated chromatin. The ChIP patterns of Tip60 in the promoter-proximal coding region of *Hoxd4* and *Gapdh* were similar to those of Lys16ac, while Mof showed distinct patterns (Figures 6F and 6G). However, in the distal coding region of *Gapdh*, the ChIP pattern of Mof was similar to that of Lys16ac (Figure 6H), while the similarity in that of Tip60 became less prominent. These results suggest that both Tip60 and Mof are the enzymes that acetylate Lys16 downstream of Ash1l-dependent Lys36me and that they differentially associate with a target gene in a region-dependent manner, i.e. Tip60 in a promoter-proximal coding region and Mof in a distal coding region. The involvement of the acetyltransferase activity of Tip60 in *Hoxd4* activation was further suggested by utilizing *Tip60* knock-in mutant ES cells (heterozygote) (Figure S9). Under these conditions, Lys36me2 was likely to be affected, suggesting crosstalk between Ash1l and Tip60.

### Interplay with Brg1, a key factor for chromatin reprogramming, is revealed by DRB

Having demonstrated functional interaction between Lys36me by Ash1l and Lys16ac by Tip60 or Mof, we then analyzed other events that the interaction influences. Among the chromatin remodeling complexes associated with gene activation, several *in vitro* studies suggest that the Brg1 complex is the most plausible candidate that targets Lys16ac [29], [30], although whether this applies *in vivo* remains unclear. The ChIP pattern of Brg1 in the promoter-proximal coding region of *Hoxd4* was mostly similar to those of Lys36me and Lys16ac (Figure 6I, left panel). We observed a similar result for *Gapdh* (Figure 6I, middle panel), even in the distal coding region (Figure 6I, right panel). Next, as the active P-TEFb complex containing both Cdk9 and Brd4 has been shown to target Lys16ac [31], we also analyzed the occupancy of Cdk9. However, the ChIP pattern of Cdk9 showed only a limited similarity to those of Lys36me and Lys16ac (data not shown). Therefore, these results suggest that Lys36me by Ash1l contributes to Brg1 association in an entire coding region.

### Ash1l is required for a proper response to a certain activating cue during development

**Figure 7:**
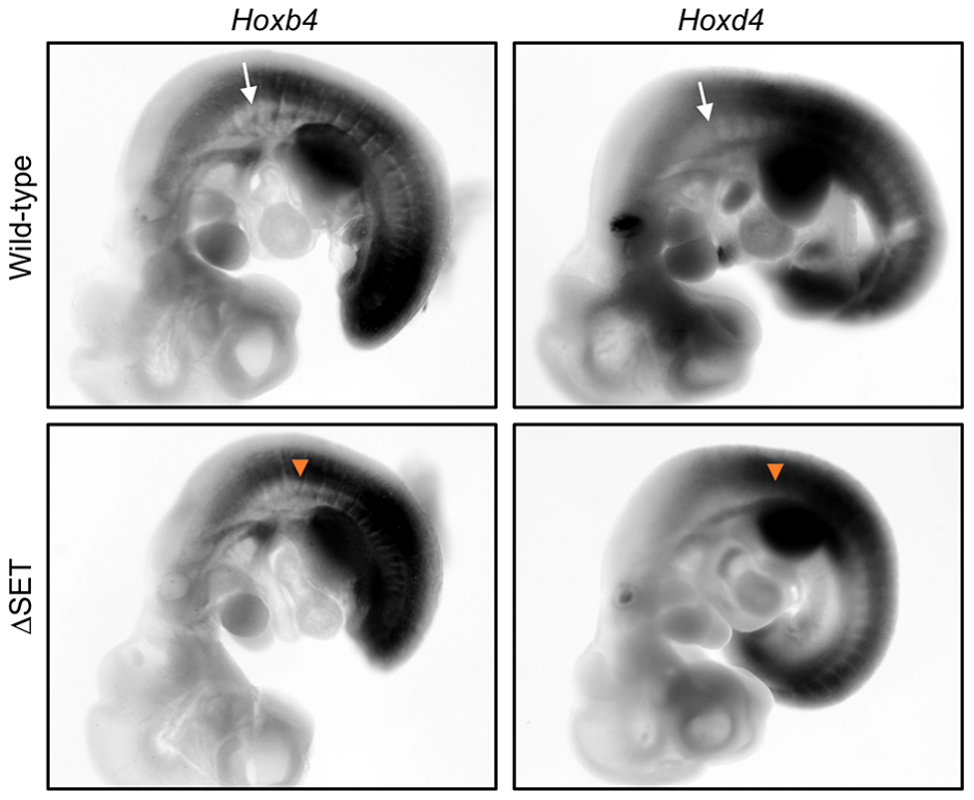
Posterior shifts of the expression boundaries of *Hoxb4* and *Hoxd4* mRNAs. Whole-mount *in situ* hybridization analyses of *Hoxb4* and *Hoxd4* mRNA expression in E10.5 embryos. Shown are normal (white arrows) and affected (orange arrow-heads) anterior expression boundaries at the paraxial mesoderm in wild-type and ΔSET mutant embryos, respectively.

We next examined whether our results in ES cells could be recapitulated in development of mice. Whole-mount *in situ* hybridization was employed to determine expression patterns of representative Hox genes in various parts of developing embryos that carry the ΔSET mutation. While the expression patterns of *Hoxb4*, *d4*, and *a4* mRNAs were largely similar between wild-type and ΔSET embryos, the anterior boundaries of their expression domains were shifted posteriorly along the antero-posterior axis at the paraxial mesoderm in ΔSET embryos (Figures 7, S10A and S10B). Thus, consistent with the results in ES cells, these findings suggest that the methyltransferase activity of Ash1l promotes a response to a certain activating cue that triggers Hox gene expression during development.

### A genetic interaction between *Ash1l* and *Mel18* in a skeletal phenotype

**Figure 8:**
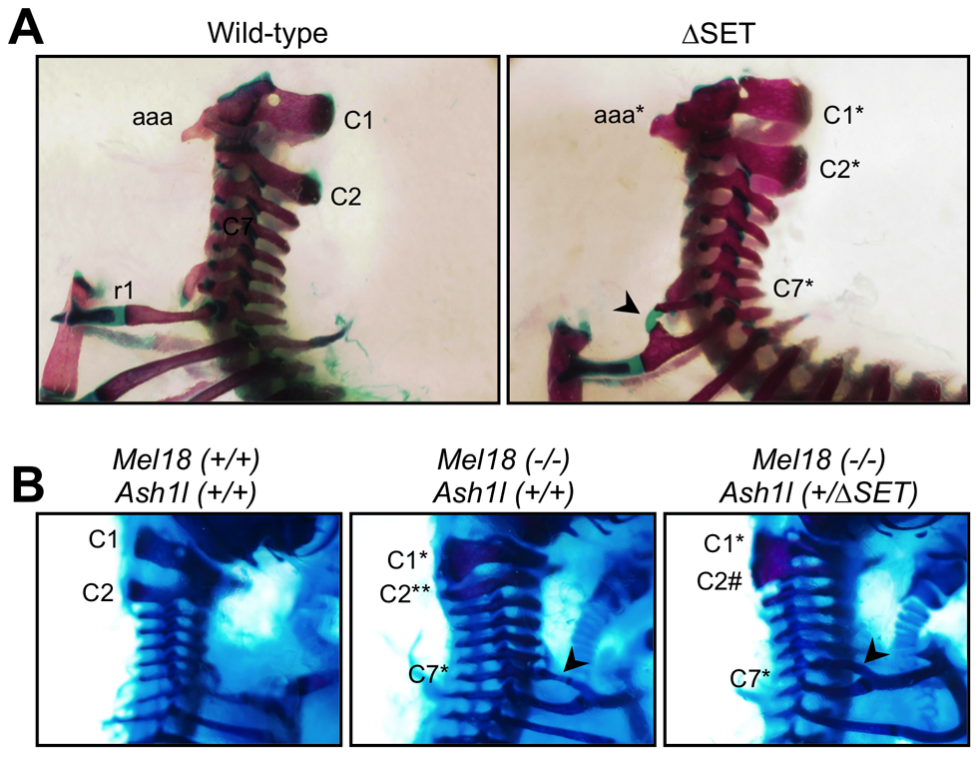
Typical skeletal phenotypes of *Ash1l* ΔSET mice, and a genetic interaction between *Ash1l* and *Mel18*. Lateral views of the cervico-thoracic region of the axial skeleton are shown. (**A**) The C2-to-C1 transformation in a ΔSET mouse (C2*), deformities of the anterior arch of the atlas (aaa* at C1*) and an ectopic rib (arrow-head) on the C7 vertebra (C7*). (**B**) A genetic interaction between *Mel18* mutant allele and *Ash1l* ΔSET allele. The C2-to-C3 transformation (C2**) in a *Mel18* mutant mouse is partially suppressed by an additional *Ash1l* ΔSET allele (C2#).

To examine whether the observed posterior shifts of the expression domains of *Hoxb4* and *Hoxd4* mRNAs are reflected by skeletal phenotype, we compared vertebrae of wild-type and mutant mice. Consistent with Ash1l being one of the trithorax group proteins, obvious alterations in the identities of vertebrae were observed (Table 1, Figures 8A and S10C–S10I). In particular, 42–56% of the mutant mice had cervical vertebrae affected, showing the homeotic transformation of the C2 vertebra into the C1 vertebra. These phenotypes were similar to those caused by mutations in group 4 Hox genes, since the C2-to-C1 transformation was caused by a functional loss of either *Hoxb4* or *Hoxd4* [32], which support our results in ES cells and embryos. Importantly, we found that the ΔSET allele partially suppressed the C2-to-C3 transformation caused by homozygous mutations in *Mel18*, indicating a role for Ash1l in anti-Polycomb silencing *in vivo* (Figure 8B).

**Table 1:** Skeletal phenotypes observed in progenies by intercrossing of heterozygotes.

Region and type of abnormalities		Genotypes		
		+/+	+/ΔSET	ΔSET/ΔSET
Cervical region				
C1	Fusion of the anterior arch of atlas to the dens of C2	0	6 (17%)	5 (20%)
	Incomplete ventral arch (right side)	0	2 (6%)	2 (8%)
C2	Broadened neural arch (C2 to C1)	0	15 (42%)	14 (56%)
C4–C7	Fusion of the neural arch (C4 and C5/C5 and C6)	0	1 (3%)	1 (4%)
	Ectopic rib from C7 (C7 to T1)	0	1 (3%)	3 (12%)
Thoracic region				
	T1 to C7	0	1 (3%)	0
	Abnormal rib cage	0	2 (6%)	1 (4%)
Lumbar region				
	L6 to S1	0	2 (6%)	0
Total number affected		0	19 (53%)	20 (80%)
Total number unaffected		12 (100%)	17 (47%)	5 (20%)
Total number analyzed		12	36	25

## Discussion

In contrast to prevalent notions, at least with regards to Hox gene activation, the present study has shown that both Lys36me2/3 in a coding region and the accompanying exclusion of the PRCs from the region occur independently of productive transcriptional elongation. RNA-Seq analysis revealed a significant functional relationship between Ash1l and Polycomb-regulated genes in that Ash1l-mediated Lys36me counteracts Polycomb silencing. Intriguingly, ChIP-Seq analysis has suggested that the preceding Lys36me2/3 during the establishment of Hox gene expression is applicable to a subset of RAR-associated genes. We have also uncovered a functional link among Ash1l, Tip60, Mof, and Brg1, which cooperatively promote Hox gene expression in response to RA. Collectively, our results reveal insights into mechanisms underlying the establishment of transcriptional memory that counteracts Polycomb silencing, which have until now been difficult to analyze by conventional methods.

**Figure 9:**
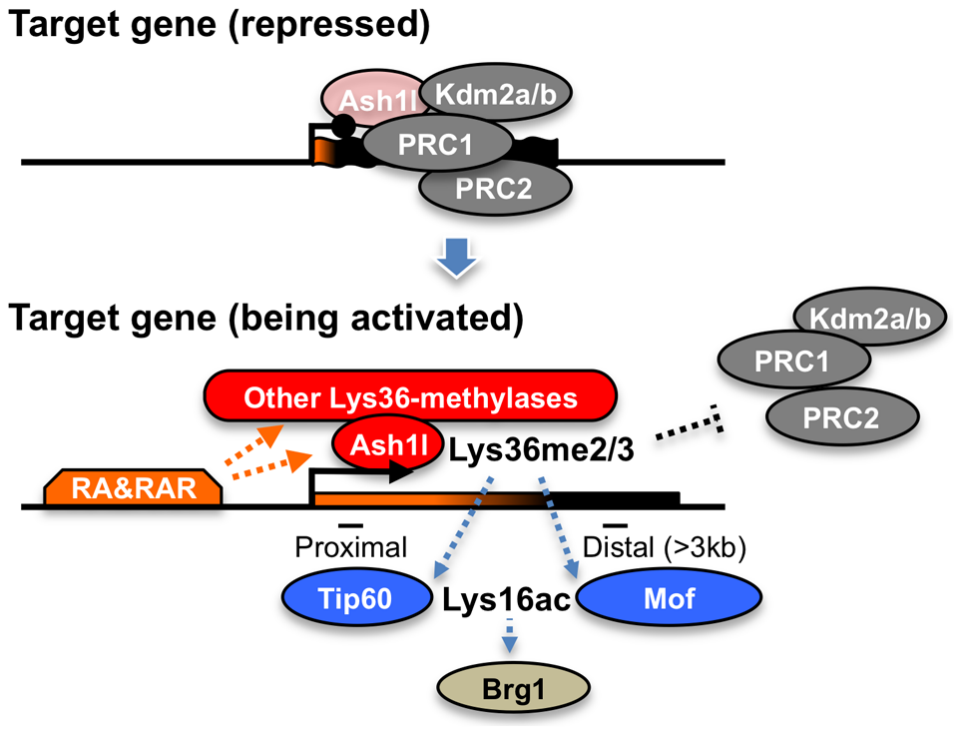
A proposed role of Ash1l with RAR in the establishment of transcriptional activation. In the upper panel, Ash1l is preloaded in the promoter-proximal coding region with the condensed bivalent chromatin (thick and short gene body) that mainly generates immature short transcripts (represented in orange on the gene body). Enzymatic activity of Ash1l is inactivated under the condition (light-red). During the establishment of transcriptional activation, retinoic acid and its receptor (RA&RAR) promote activation of Ash1l (dark-red), as well as association of the other Lys36-methylases with the target chromatin. These Lys36-methylases, including Ash1l, orchestrate the downstream mechanisms directly or indirectly, thereby further promoting RA response through alleviating the repressive effect of the PRCs and opening the condensed chromatin (represented by the extended shape of the gene body in the bottom panel) independently of transcriptional elongation. The Brg1 complex may indirectly target Lys36me2/3 through Lys16ac.

Here, we propose that Ash1l and RAR coordinate to orchestrate a novel regulatory cascade of chromatin reprogramming (Figure 9). The Lys36me2/3 preceding productive transcriptional elongation may directly counteract association of the PRCs in target chromatin [33], [34], resulting in de-repression from Polycomb silencing, likely through loosening of the compacted chromatin structure [35]. Therefore, Lys36me2/3 by Ash1l and other Lys36-methylases constitute a rate-limiting step, which may promote Lys16ac by Tip60 or Mof in a region-dependent manner. Lys16ac may lead to further loosening of the chromatin structure [29], allowing the Brg1 chromatin remodeling complex to be associated and to promote chromatin reprogramming, presumably by further excluding the PRCs to alleviate Polycomb silencing and by remodeling nucleosomes to facilitate productive transcriptional elongation.

The proposed mechanism might be also applied to transcriptional regulation in the *Drosophila* species, showing a correlation between Lys36me2 and Lys16ac [24]. Indeed, the consecutive regulatory steps described above might explain a previous report detailing progression of the ecdysone-induced puff 74EF in polytene chromosomes of *Drosophila* larvae under pretreatment with DRB [36]. However, the mechanism would not apply in yeast, in which an anti-correlation between Lys36me2 and histone H4 acetylation has been reported [20], [21]. These observations suggest that such regulatory mechanisms are unique to metazoans.

What is the significance of Lys36me3 during the establishment of transcriptional activation? At least in a promoter-proximal coding region of *Hoxd4*, we found Lys36me3 could occur independently of productive transcriptional elongation (Figure 5C). An accumulation of Lys36me3 on the Lys36me2-platform may ensure de-repression from Polycomb silencing because Lys36-demethylases Kdm2a/b would not recognize Lys36me3 as a substrate [33]. The degree of Lys36-trimethylase recruitment was presumably RA-dependent as we observed only a small increment in the level of Lys36me3 in the presence of DRB in B16 cells without addition of RA (Figure S5). A predisposition to underrepresent RAR-associated genes in the “decreased” gene groups in response to DRB as well as accumulations of Lys36me2/3 around RAR binding sites further support our surmise (Figures 4G and 4H). Of note, DRB clearly increased the Lys36me2/3 levels in promoter-proximal coding regions of *Hoxb4/d4* in ΔSET ES cells (Figures 4A and S4B). Therefore, we speculate that several Lys36-methylases, including Ash1l, play a role during the establishment of transcriptional activation in an RA-dependent manner. Consistent with this speculation, Ash1l, Nsd1, and a major mammalian Lys36-trimethylase Setd2, all have a nuclear receptor binding motif, L*XX*LL. Indeed, approximately 60% of RAR binding sites were co-occupied with Ash1l (Figure S1G). Thus, it is tempting to speculate that nuclear receptor-dependent developmental programs may have similar underpinnings to the Hox genes regulator mechanisms revealed in this study.

Our results suggest that a part of the function of Lys36me2/3 in *Hoxd4* mRNA expression is masked after productive transcriptional elongation. Specifically, the effect of Lys36me2/3 on the association with Tip60 and Brg1 was more evident in the presence of DRB (Figures 6F–6I), suggesting that this association is partially dependent on P-TEFb activity. Once the active P-TEFb complex associates with target chromatin and triggers the productive transcriptional elongation, it may have a dominant effect on the association over that of Lys36me2/3. However, upon gene activation but before tethering of the P-TEFb complex, Lys36me2/3 may have a dominant comprehensive function, involving exclusion of the PRCs and promoting association of Tip60 and Brg1, thereby facilitating the RA response (Figure 9). This idea is consistent with our results demonstrating that sensitivity against a certain activating cue appeared to be affected in ΔSET mice and ES cells (Figures 1F, 7, S10A and S10B).

One of the important issues when studying transcription mechanisms on a chromatin template is how a dramatic change in chromatin structure occurs upon gene activation: in particular, whether the open chromatin structure is established before or after the first RNAPII travels along the template DNA [37]. So far, it is widely believed that a specially equipped RNAPII, or so-called “pioneer polymerase”, is required for the initial opening of the condensed chromatin. This special RNAPII breaks down the condensed chromatin structure into the open structure during the first transcriptional elongation, thereby ultimately creating the transcription-competent chromatin. However, the results of the present study led us to the notion that the driving forces initiated by the methyltransferase activity of Ash1l promote the establishment of the open and transcription-competent chromatin structure prior to the first productive transcriptional elongation by fully-activated RNAPII. Our hypothesis may be applied to active but non-productive bivalent genes; however, it remains unclear whether it can be applied to inactive, inducible monovalent genes.

Results from whole-mount *in situ* hybridization analyses in *Ash1l* ΔSET mice (Figures 7, S10A and S10B) were clearly distinct from those in mutant mice carrying a deletion in the SET domain of Mll1, a representative Lys4-methylase belonging to the trithorax group, which displayed a normal expression boundary and an impaired maintenance of *Hoxd4* mRNA expression [38]. On the other hand, the results in *Ash1l* ΔSET mice were similar to those in the Polycomb group mutant mice in that the mutants demonstrated shifts of expression boundaries at the paraxial mesoderm (*Mel18* in [39]; *Phc1/2* in [40]), although directions of the shifts in Polycomb group mutant mice were opposite to those in *Ash1l* ΔSET mice. Collectively, these results suggest that Ash1l has a distinct function from Mll1 and directly counteracts the function of the Polycomb group proteins. Consistent with this idea, *Ash1l* ΔSET mice only demonstrated additive and non-synergistic phenotypes with the double-heterozygous *Mll1* mutation [YY & KN, unpublished observation], and a partial suppression in the phenotype with the *Mel18* mutation (Figure 8B).

We also observed that Ash1l was localized in a promoter-proximal coding region (Figures 3E, 3F and S1E), corroborating previous reports [8], [9]. Bromo-, PHD- and BAH-domains in the carboxyl-terminal region of Ash1l supposedly function to restrict localization. The distribution of Ash1l in *Hoxd4* was similar to those of Lys4me2/3, and a large portion of Ash1l was co-localized with Lys4-methylated chromatin (Bivalent and Lys4me alone, Figure S1F). It is tempting to speculate that the specific localization of Ash1l may be necessary for certain interaction partners of Ash1l, such as Ly4-trimethylases, to be recruited in a promoter-proximal coding region. Of note, we also found that Ash1l was clearly present in the absence of RA (Figure 3E) and in genes that were not expressed (Figures S1F and S1G). Surprisingly, it appeared that the methyltransferase activity of Ash1l was inactive under these conditions. Presumably, Ash1l is deposited but poised to achieve an immediate action in response to RA. It remains unclear how the enzymatic activity of Ash1l protein is activated. Future studies on the Ash1l complex and its interaction partners, as well as using knockout mice, may resolve these issues.

Unexpected is the increase in the level of Lys36me2 upon DRB treatment. It is possible that, under normal conditions, there may be a competition for methylation sites between Lys36-trimethylase Setd2 and other Lys36-dimethylases including Ash1l. In the presence of DRB, the lack of transcription-dependent trimethylation by Setd2 would result in a spreading of Lys36me2 catalyzed by the dimethylases. In a subset of RAR-associated genes, the Lys36-trimethylase, accompanied with RAR, may generate Lys36me3 on the plat-form of accumulated Lys36me2 in a transcription-independent manner. This may explain the increased levels of Lys36me2/3 upon DRB treatment in the subset of RAR-associated genes including *Hoxd4*.

In this study, using an Ash1l mutant and DRB, we have revealed a novel function for Ash1l during the establishment of transcriptional activation of Polycomb-regulated genes, including Hox and Wnt family genes. Given that the Wnt signaling pathway integrates numerous environmental signals *in vivo*, Ash1l may modulate a variety of signals in many biological processes. We have also found novel functional links among several chromatin modifiers that reprogram the status of target chromatin. Future studies on these factors will provide further insights into precise mechanisms for the establishment of transcriptional memory that counteracts Polycomb silencing of developmentally regulated genes.

## Materials and Methods

### Ethics statement

The animals' care was in accordance with institutional guidelines of National Institute of Genetics in Japan and Saga University Faculty of Medicine.

### Generation of *Ash1l* ΔSET mice

The schematic representation of the strategy used for targeted disruption of mouse *Ash1l* gene is shown in Figure 1A. A targeting vector was constructed by insertion of DNA fragments of introns 10–12 (5′SphI-SpeI) of mouse *Ash1l* gene into a ploxFNFDT-SS backbone vector, in which 5′BamHI-3′SphI fragment was replaced to a PCR-cloned floxed exon fragment (exons 11–12) with a Pgk-Neo^r^ cassete. PCR primer-pairs used for the cloning are listed in Table S4. ΔSET mice were generated with M1 ES cells (derived from *F1* of C57BL/6J and 129/Sv), and backcrossed to C57BL/6J between two to six times. Genotypes were determined by PCR using the primer-pairs listed in Table S4.

### Generation and characterization of polyclonal antibodies against mouse Ash1l protein

cDNA encoding a part of Ash1l protein (2803–2891, Figure S1C) was inserted into the bacterial expression vector pGEX 6P-1 (GE Healthcare). The PCR primer-pairs used are listed in Table S4. GST-fusion proteins were induced and were purified using glutathione-sepharose beads. The eluates containing the recombinant proteins were pooled and dialyzed against PBS. The antibodies were raised against each GST-fusion protein and affinity-purified. Since endogenous Ash1l protein was difficult to detect by immunoblot, the specificity of the antibodies was checked by immunofluorescence analysis under transient expression of lentiviral-mediated shRNA directed against mouse *Ash1l* mRNA (Figure S1D). Pseudovirus was produced from HEK293T cells by cotransfection of packaging plasmids (Addgene) and pRSI9 vector (Cellecta, Decipher Project) using PEI-MAX (Polysciences). The target sequence in *Ash1l* mRNA was following: 5′-GCCAAAUUCUCCUUCUCAUUU-3′.

### Cell cultures

ES cells were cultured on gelatin-coated dishes in a basic culture medium of KO-DMEM (Gibco) containing 1× GlutaMAX-I (Gibco), 1× MEM NEAA (Gibco), 0.1 mM 2-mercaptoethanol (Gibco), 50 units/ml penicillin (Gibco), 50 µg/ml streptomycin (Gibco), without feeder cells. For culturing undifferentiated ES cells, the above basic culture medium was supplemented with 1,000 units/ml of leukemia inhibitory factor (LIF) (Chemicon), 15% Knockout Serum Replacement (Invitrogen), and 1% fetal calf serum (Gibco), and 10 mM 4-(2-hydroxyethyl)-1-piperazineethanesulfonic acid (Hepes) buffer. For culturing differentiating ES cells, only 10% fetal calf serum (Gibco) was supplemented to the above basic culture medium. A typical protocol for cell culture is shown in Figure 1D, in which RA is added to the differentiation medium at indicated time points. DRB (Sigma) was added at a final concentration of 75 µM on either Day 3 or Day 4 (16 hour-exposure) before analysis.

### ChIP assays

Chromatin immunoprecipitation was performed according to online protocols provided by Millipore (for histone modifications) or Abcam (for the other proteins) with modifications in fixation protocols. The antibodies and fixation protocols used are listed in Table S5. Immunoprecipitated DNA was purified using a PCR Purification Kit (Qiagen), and was quantified by real-time PCR using SYBR green dye on a LightCycler480 machine (Roche). PCR temperatures for acquisitions of DNA amplification signals were determined empirically. PCR primer-pairs used are listed in Table S4. Background signals are shown in Figure S11A and are subtracted from most of the respective results. Control ChIP signals in either a promoter-proximal coding region of *Gapdh* or a promoter region of *Il2ra* are indicated in relevant figures (Figure S11B). Unless otherwise stated, each result and error bar in graphs represent mean and s.d., respectively, of three independent PCR reactions from a single ChIP experiment that is representative of several that were performed (3 to 5 experiments).

### ChIP-Seq and data analysis

For ChIP-Seq, 1–5×10^7^ ES cells were used and chromatin was sheared to an average DNA fragment size of 150–250 bp. After immunoprecipitation using Dynabeads protein G (Invitrogen), ChIP-Seq libraries were prepared according to Illumina protocols. The libraries were sequenced using an Illumina HiSeq 1000. All ChIP-Seq reads were mapped to the mouse genome (mm9) using Bowtie2 with default parameters. Genomic profiles were generated using igvtools and were viewed in Integrative Genomics Viewer (IGV). Peaks of Ash1l and RAR ChIP-Seq signals on genome were determined using MACS2 with false-discovery rate as 0.05. Each associated gene for the peaks was determined using Entrez gene annotation with in-house computer program, in which Ash1l-target genes were defined as genes containing Ash1l-peaks around transcription start site (TSS) within +/−4 kb and RAR-associated genes were defined as genes containing RAR-peaks in up-stream (from −20 kb to TSS) and coding regions. Datasets for reads/kb/million mapped (RPKM) values of Lys36me2/3 in coding regions of each gene were normalized to 75th percentile. Raw sequencing data were submitted to the NCBI Short Read Archive database under accession number (GSE48421). Mouse ES cell RAR ChIP-Seq datasets (GSE19409) [22] were downloaded from the NCBI Short Read Archive database and were compared with Lys36me2/3 datasets generated by our study.

### *In situ* RNA hybridization

Hox cDNAs were RT-PCR-cloned from embryonic total RNA into pBluescript. Primer-pairs used for PCR amplification are listed in Table S4. Single-stranded RNA probes labeled with either [^35^S]-UTP (for section) or digoxigenin-UTP (for whole-mount) were synthesized according to manufacturer's instructions (Promega; Roche). *In situ* hybridization was performed according to the procedures described previously [41], [42]. After hybridization and washing, the sections were immersed in Kodak NBT emulsion (diluted 1∶1 with 2% glycerol), exposed for 2 weeks and developed in a Kodak D-19 developer. For whole-mount *in situ* hybridization, probes were detected using alkaline phosphatase-conjugated anti-digoxigenin Fab fragment (Roche) and signals were developed using Nitro blue tetrazolium chloride (NBT) and 5-Bromo-4-chloro-3-indolyl phos- phate, toluidine salt (BCIP) (Roche).

### Skeletal analysis

Skeletal preparations were prepared from perinatal mice as described previously [41]. Cartilage and ossified bone were stained with alcian blue-alizarin red.

### Nuclear run-on assays

The run-on transcription assay was performed as described previously with following modifications [43]. Briefly, 5–7×10^6^ cells were treated with ice-cold hypotonic nuclei isolation buffer (20 mM Hepes-KOH [pH 7.6], 10 mM NaCl, 5 mM MgCl_2_, 0.5% NP-40, 1 mM DTT, 0.2 mM PMSF, 1 mM Bezamidine-HCl) and the isolated nuclei were re-suspended in storage buffer (50 mM Hepes-KOH [pH 7.6], 0.1 mM EDTA, 5 mM MgCl_2_, 40% glycerol) to give a total 30 µl for each reaction. Transcription was re-started by addition of 30 µl of transcription buffer (10 mM Hepes-KOH [pH 7.6], 0.3 M KCl, 4 mM DTT), 40 units of RNase inhibitor, 3 µl of Biotin RNA Labeling Mix (Roche). The reaction was incubated at 30°C for 45 min on a vortex mixer. After DNase I (Takara) treatment, total RNA was isolated using Isogen II (Nippongene) and 10–20 µg of total RNA was subjected to further purification of nascent RNA molecules using 50 µl of Dynabeads MyOne Streptavidin T1 (Invitrogen) in Click-iT Nascent RNA Capture Kit (Invitrogen). Complementary DNAs were synthesized from purified nascent RNA molecules by on-beads reverse transcription according to the manufacturer's instructions, and the cDNAs were subjected to real-time PCR analyses.

### RNA-Seq and data analysis

Total RNA was prepared using Isogen II (Nippongene) and subjected to DNase I (Takara) treatment and further purified by aid of RNeasy Mini Kit column (Qiagen). The poly(A)-containing mRNA were purified and libraries were prepared according to Illumina TruSeq RNA protocols. Data were obtained with the Illumina HiSeq 1000 sequencing machine. All RNA-Seq reads were mapped to the mouse genome (mm9) using TopHat2. Transcript abundance was quantified using Cufflinks and annotations from Ensembl release 70, and FPKM (fragments/kb of transcript/million fragments mapped) values were calculated. To minimize dispersion effect by low-FPKM values, all the FPKM values were modified by addition of 0.1 in log2 transformation. For a classification of chromatin signature, a supplementary table and ChIP-Seq data in Mikkelsen, et al. [18] were used as references. Gene ontology analysis for biological process of the selected genes was performed using Partek Genomic Suite (Ryoka systems). Raw sequencing data were submitted to the NCBI Short Read Archive database under accession number (GSE48419).

Remaining materials and methods including the method for histone methyltransferase assay are available in Text S1.

## Supporting Information

Figure S1 Characterization of *Ash1l* gene product (mRNA expression and genomic distribution of Ash1l protein). (**A**) Northern blot analysis of *Ash1l* mRNA expression using total RNA from various adult tissues and whole embryos. (**B**) Conventional RT-PCR analyses of *Ash1l* mRNA expression levels in undifferentiated or differentiated ES cells, developing embryos (E8.5, 10.5, 14.5) and embryonic fibroblasts (MEFs). As controls, expression levels of *Oct4*, *Hoxd4*, and *Gapdh* mRNAs are shown. After RA was added to the culture medium at a final concentration of 1 µM in the absence of LIF and feeder cells, ES cells were further cultured for 4 days. (**C** and **D**) Characterization of the antibodies against Ash1l protein. Rabbit polyclonal antibodies were raised against the carboxyl-terminal region of mouse Ash1l protein (an arrow in C, see Materials and Methods). Immunofluorescence analysis of Ash1l protein in mouse embryonic fibroblasts (D). A lentivirus vector expressing shRNA directed against *Ash1l* mRNA was constructed, and a recombinant virus was infected to mouse embryonic fibroblasts. The virus-infected fibroblasts were labeled by TagRFP. Nuclei were labeled by DAPI. The empty vector was used as a shRNA-negative control. (**E**) Distribution of Ash1l ChIP-Seq read counts relative to TSS in ES cells. (**F**) Pie chart showing relative ratio of status of chromatin signatures [18] for Ash1l-target genes. (**G**) Venn diagrams showing the relationship of Ash1l-target genes with either Lys4me-positive genes [Lys4me3 (+)], expressed genes (Expressed, FPKM values from RNA-Seq analysis over 0.1), or RAR-associated genes [RAR (+)]. The numbers of genes in each compartment are shown. The total number of annotated genes analyzed was 18,724.
(TIF)

Figure S2 RNA-Seq data for Hox and Wnt family genes in differentiating ES cells. (**A** and **B**) The results of Hox (A) and Wnt (B) family genes were plotted on the graphs using modified FPKM values. The x-axis corresponds to expression levels of each gene (shown as log2 transformation of each FPKM value plus 0.1), and the y-axis corresponds to fold change in gene expression levels between ΔSET ES cells and wild-type (shown as Δlog2 transformation). (**C**) Quantitative RT-PCR analyses of *Hoxd4*, *Wnt6*, and *Gapdh* mRNAs in differentiating ES cells to verify the RNA-Seq results.
(TIF)

Figure S3 RNA-Seq data for marker gene expression. The results of indicated marker genes are plotted on the graphs using modified FPKM values. The x-axis corresponds to expression levels of each gene (shown as log2 transformation of each FPKM value plus 0.1), and the y-axis corresponds to fold change in gene expression levels between ΔSET ES cells and wild-type cells (shown as Δlog2 transformation). (**A**) Undifferentiated ES cells. (**B**) Differentiating ES cells.
(TIF)

Figure S4 ChIP assays of histone modifications for *Hoxb4* and *Hprt1* in differentiating ES cells. (**A**) Diagrams of *Hoxb4* and *Hprt1* genes. Black boxes represent exons. (**B**–**D**) ChIP assays of histone modifications and the status of RNAPII in differentiating ES cells either with (+) or without (−) DRB treatment. The antibodies used are indicated at the top of each graph. The results are represented as means and s.d. (B) The promoter-proximal coding region of *Hoxb4* was analyzed. (C) The promoter-proximal coding region of *Hprt1* was analyzed. (D) The distal coding region of *Hprt1* was analyzed.
(TIF)

Figure S5 Comparison of DRB-response between ES cells and B16 cells. (**A**) Quantitative RT-PCR analyses of *Hoxd4* and *Gapdh* mRNAs in differentiating ES cells and a melanoma cell line, B16. ES cells were cultured in the presence of RA (see the culture protocol shown in Figure 1D). *Hoxd4* was constitutively active in B16 cells without addition of RA. The left panel depicts expression of *Hoxd4* mRNA in the presence (+) or absence (−) of DRB. The right panel depicts expression of *Gapdh* mRNA. The results are represented as the means and s.d. of three independent PCR reactions. (**B**) RA-dependent increases in Lys36me2/3 levels of *Hoxd4* chromatin in response to DRB. ChIP assays of B16 cells and differentiating ES cells either with (green bars) or without (black bars) DRB treatment. The promoter-proximal coding region of *Hoxd4* in each cell was analyzed. The antibodies used are indicated at the top of each graph. The results are represented as means and s.d.
(TIF)

Figure S6 Exclusion of the PRCs occurs in a transcription-independent manner. ChIP assays of Lys27me3 and Mel18 in differentiating ES cells either with (+) or without (−) DRB treatment. (**A**) Occupancies of Lys27me3 and Mel18 in the promoter-proximal coding region of *Hoxd4* before addition of RA. (**B** and **C**) DRB was added to the culture medium prior to RA, resulting in induction over 16 hours. The promoter-proximal (B) and distal (C) coding regions of *Hoxd4* were analyzed. In (B), the same dataset as in Figure 5D was used. (**D**) RA was added to the culture medium prior to DRB as shown in Figure 1D, resulting in induction over 48 hours. The antibodies used are indicated at the top of each graph. The results are represented as means and s.d.
(TIF)

Figure S7 The status of RNAPII is mostly unaffected in ΔSET ES cells. (**A** and **B**) ChIP assays of various regions of *Hoxd4* in differentiating ES cells before (A) or after (B) addition of RA. The results are represented as relative values that were obtained by normalizing each result to *Gapdh* in each cell type. Error bars represent s.d. of three independent ChIP experiments. The antibodies used are indicated above each graph. Broken lines show approximate levels of ChIP signals in the *Il2ra* promoter. We found that RNAPII was relatively enriched in the promoter-proximal region even before *Hoxd4* activation (A), demonstrating one of the features of promoter-proximal pausing of the poised RNAPII. After RA treatment, the RNAPII levels in the coding regions were increased in both wild-type and ΔSET ES cells to a similar extent (B), suggesting that the recruitment and progression of RNAPII were not affected in ΔSET ES cells. Similar results were obtained with the phosphorylation levels of Ser2 (Ser2p) and Ser5 (Ser5p) at the carboxyl-terminal domain of RNAPII; however, the Ser2p levels in the coding regions of ΔSET ES cells were observed to be slightly affected (B). (**C**) A diagram of the *Hoxd4* gene. Black and grey boxes represent exons and a 3′ RARE, respectively. Black bars under the diagram indicate the regions analyzed by ChIP assays. TSS: transcription start site. (**D**) ChIP assays of promoter-proximal and distal coding regions of *Wnt6* in differentiating ES cells. The results are represented as relative values that were obtained by normalizing each result to *Gapdh* in each cell type. Error bars represent the s.d. of three independent ChIP experiments. The antibodies used are indicated above each graph. Broken lines show approximate levels of ChIP signals in the *Il2ra* promoter. (**E**) A diagram of the *Wnt6* gene. Black boxes represent exons. Black bars under the diagram indicate the regions analyzed by ChIP assays.
(TIF)

Figure S8 ChIP assays of histone H4 Lys16 acetylation for *Hoxb4* and *Hprt1* in differentiating ES cells. Diagrams of *Hoxb4* and *Hprt1* genes are shown on top of each ChIP result. Black boxes represent exons. ChIP assays were performed using differentiating ES cells either with (+) or without (−) DRB treatment. The results are represented as means and s.d. (**A**) The promoter-proximal coding region of *Hoxb4* was analyzed. (**B**) The promoter-proximal (left) and distal (right) coding region of *Hprt1* was analyzed.
(TIF)

Figure S9 Generation of *Tip60* knock-in mutant ES cells. (**A**) Schematic representation of the strategy used for targeted replacement of exon 10 in the *Tip60* gene. The mutated exon 10 encoding a part of the histone acetyltransferase domain with its flanking introns was floxed by loxP sequences. FLPe-mediated recombination resulted in the generation of the mutated allele (Q325E and G328E, heterozygote). Red bars represent mutations in exon 10. (**B**) RT-PCR analysis of RA-induced *Hoxd4* mRNA expression. (**C**) ChIP assays of histone modifications in differentiating ES cells either with (+) or without (−) DRB treatment. The promoter-proximal coding region of *Hoxd4* was analyzed. The antibodies used are indicated at the top of each graph. The results are represented as means and s.d. Likely due to the heterozygosity of the knock-in mutation, we observed a mild difference in the levels of Lys16ac between wild-type and knock-in mutant ES cells.
(TIF)

Figure S10 *In vivo* analyses of *Ash1l* ΔSET mutant mice. (**A** and **B**) In situ hybridization analysis of *Hoxa4* mRNA in E11.5-embryos. In (A), results of whole-mount in situ hybridizations for *Hoxa4* mRNA are shown. Normal (white arrows) and affected (orange arrowheads) anterior expression boundaries at the paraxial mesoderm in wild-type and ΔSET embryos. In (B), results of in situ hybridizations for *Hoxa4* mRNA are shown in a representative cross-sectional image. A radio-isotope-labeled antisense-riboprobe was used for the detection of the mRNA. Yellow lines represent boundaries between each pre-vertebra (pv). Each arrow indicates the most anterior boundaries of *Hoxa4* mRNA expression. An atrial chamber of the heart in each embryo is encircled by a blue-broken line. (**C**–**H**) Typical skeletal phenotypes of *Ash1l* ΔSET mice. Ventral views of the axial skeleton are shown. (C, D and E) Wild-type, (F, G, and H) ΔSET mice. (**C** and **F**) The cervical region. In (F), the dens of the C2* is fused to the C1*, affecting the formation of the anterior arch of atlas (aaa*). (**F** and **G**) The thoracic region. In (G), the abnormal rib cage is shown. Identities of sternoclavicular joints are mismatched between the left and right sides (for example, r2 to r1*). (**E** and **H**) The lumbo-sacral region. In (H), the transverse process of the L6* is fused to that of the S1. (**I**) Schematic representation summarizing the homeotic transformations. The vertebrae are numbered serially from the C1 vertebra, in which the cervical region is from 1 to 7, the thoracic region is from 8 to 20, the lumbar region is from 21 to 26, and the sacral region is from 27 to 30. a, the C2-to-C1 transformation. b, the C7-to-T1 transformation. c, the T1-to-C7 transformation. d, the L6-to-S1 transformation.
(TIF)

Figure S11 Background and control signals in ChIP assays. (**A**) Background ChIP signals in the promoter-proximal regions of indicated genes with (+) or without (−) DRB treatment are shown. The fixatives that were used are indicated above each graph (see Table S5 for fixation protocols). The results are represented as the means. Most background ChIP signals were around 0.01% input and are subtracted from most of the respective results. (**B**) Control ChIP signals in either a promoter-proximal coding region of *Gapdh* (for Lys27me3, Mel18, Suz12, H2Aub, and Rnf2) or a promoter region of *Il2ra* (for the others). ChIP assays were performed using indicated antibodies and approximate levels of each result are indicated in relevant figures.
(TIF)

Table S1 Results from intercrossing of Ash1l ΔSET heterozygote. Each offspring obtained by mating heterozygotes was genotyped around 3 to 4 weeks after birth by allele-specific PCR using the primers listed in Table S4.
(XLSX)

Table S2 RA-responsive genes and ΔSET-impaired genes. Listed are 543 genes with a value over 2.5 following Δlog2 transformation of modified FPKM values in wild-type ES cells after 10 nM RA treatment for 2 days (WT+RA) over those of undifferentiated cells (WT). Raw FPKM values are shown here. Genes that were down-regulated in ΔSET ES cells are indicated as “Yes” in column F.
(XLSX)

Table S3 Dys-regulated genes in undifferentiated ES cells. Listed are 116 genes that showed changes in expression levels in undifferentiated wild-type and ΔSET ES cells. Raw FPKM values are shown here. Genes that were down-regulated in ΔSET ES cells are indicated as “Down” (59 genes), while genes that were up-regulated in the cells are indicated as “Up” (57 genes) in column D.
(XLSX)

Table S4 Oligonucleotides used in this study. PCR primers and their sequences are listed. All oligonucleotides were synthesized by Hokkaido System Science Co., Ltd.
(XLSX)

Table S5 Antibodies used in this study. These antibodies were used for immunoblots and ChIP assays, in which amounts used for each experiment were empirically determined. Fixative conditions for ChIP assays are shown in column C.
(XLSX)

Text S1 Supplemental Materials and Methods. Remaining materials and methods are described here.
(DOCX)
