# Supplementary figures and images for: Ash1l Methylates Lys36 of Histone H3 Independently of Transcriptional Elongation to Counteract Polycomb Silencing
Source: PLoS Genet. 2013 Nov 7;9(11):e1003897. doi: 10.1371/journal.pgen.1003897 (PMC3820749; doi:10.1371/journal.pgen.1003897)

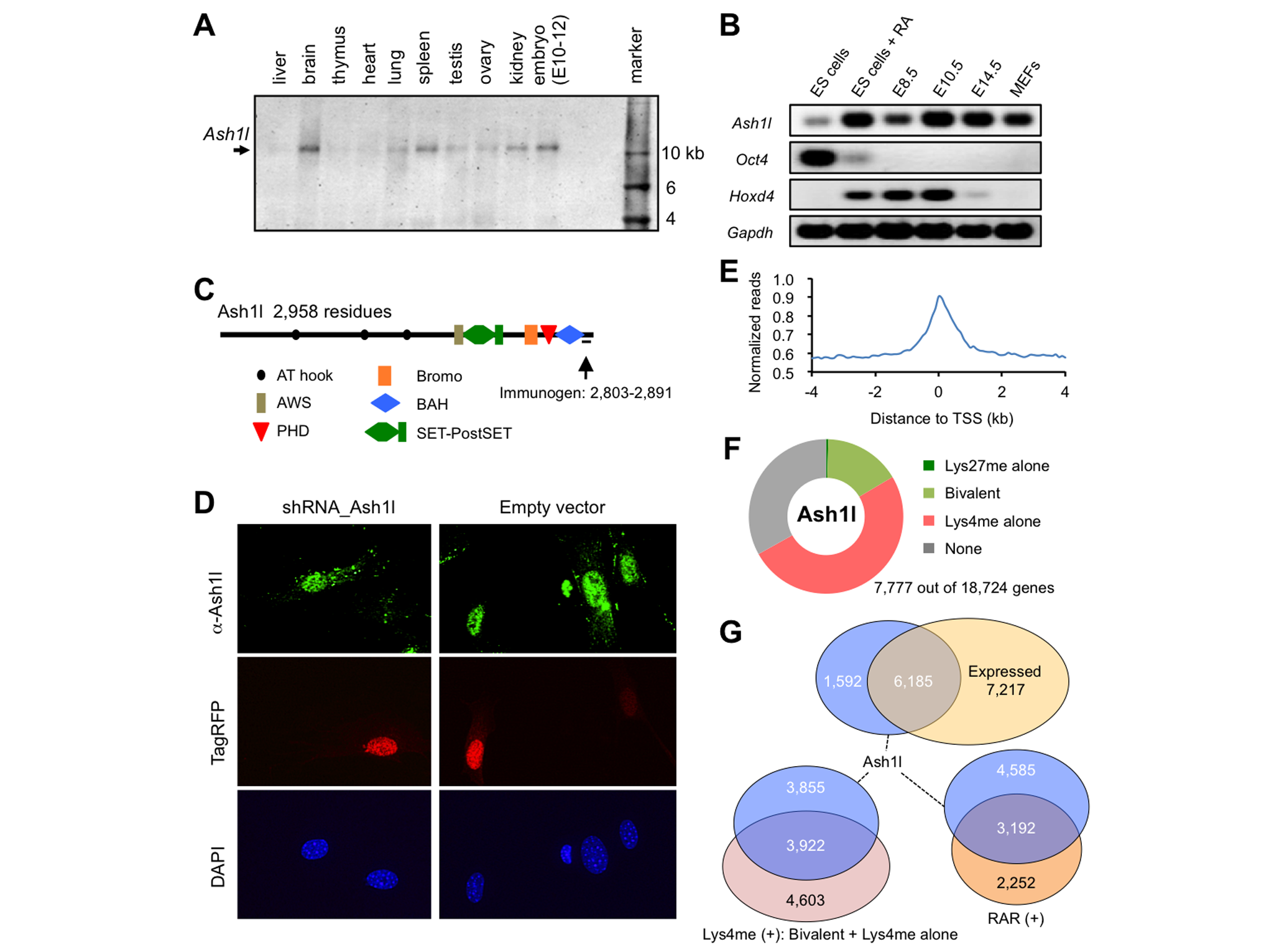

Supplement: Figure S1 — Characterization of Ash1l gene product (mRNA expression and genomic distribution of Ash1l protein). (A) Northern blot analysis of Ash1l mRNA expression using total RNA from various adult tissues and whole embryos. (B) Conventional RT-PCR analyses of Ash1l mRNA expression levels in undifferentiated or differentiated ES cells, developing embryos (E8.5, 10.5, 14.5) and embryonic fibroblasts (MEFs). As controls, expression levels of Oct4, Hoxd4, and Gapdh mRNAs are shown. After RA was added to the culture medium at a final concentration of 1 µM in the absence of LIF and feeder cells, ES cells were further cultured for 4 days. (C and D) Characterization of the antibodies against Ash1l protein. Rabbit polyclonal antibodies were raised against the carboxyl-terminal region of mouse Ash1l protein (an arrow in C, see Materials and Methods). Immunofluorescence analysis of Ash1l protein in mouse embryonic fibroblasts (D). A lentivirus vector expressing shRNA directed against Ash1l mRNA was constructed, and a recombinant virus was infected to mouse embryonic fibroblasts. The virus-infected fibroblasts were labeled by TagRFP. Nuclei were labeled by DAPI. The empty vector was used as a shRNA-negative control. (E) Distribution of Ash1l ChIP-Seq read counts relative to TSS in ES cells. (F) Pie chart showing relative ratio of status of chromatin signatures [18] for Ash1l-target genes. (G) Venn diagrams showing the relationship of Ash1l-target genes with either Lys4me-positive genes [Lys4me3 (+)], expressed genes (Expressed, FPKM values from RNA-Seq analysis over 0.1), or RAR-associated genes [RAR (+)]. The numbers of genes in each compartment are shown. The total number of annotated genes analyzed was 18,724. (TIF) [file pgen.1003897.s001.tif]

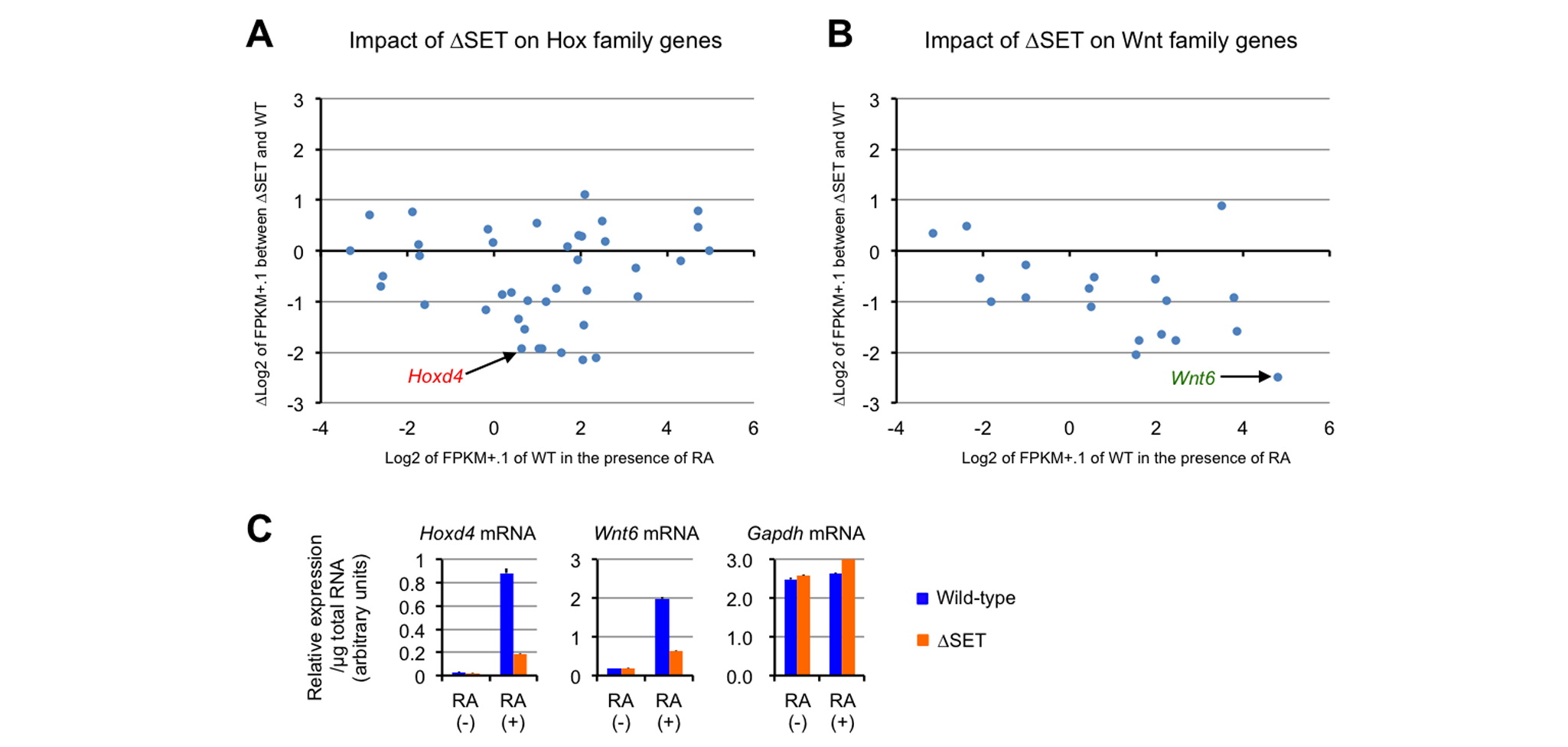

Supplement: Figure S2 — RNA-Seq data for Hox and Wnt family genes in differentiating ES cells. (A and B) The results of Hox (A) and Wnt (B) family genes were plotted on the graphs using modified FPKM values. The x-axis corresponds to expression levels of each gene (shown as log2 transformation of each FPKM value plus 0.1), and the y-axis corresponds to fold change in gene expression levels between ΔSET ES cells and wild-type (shown as Δlog2 transformation). (C) Quantitative RT-PCR analyses of Hoxd4, Wnt6, and Gapdh mRNAs in differentiating ES cells to verify the RNA-Seq results. (TIF) [file pgen.1003897.s002.tif]

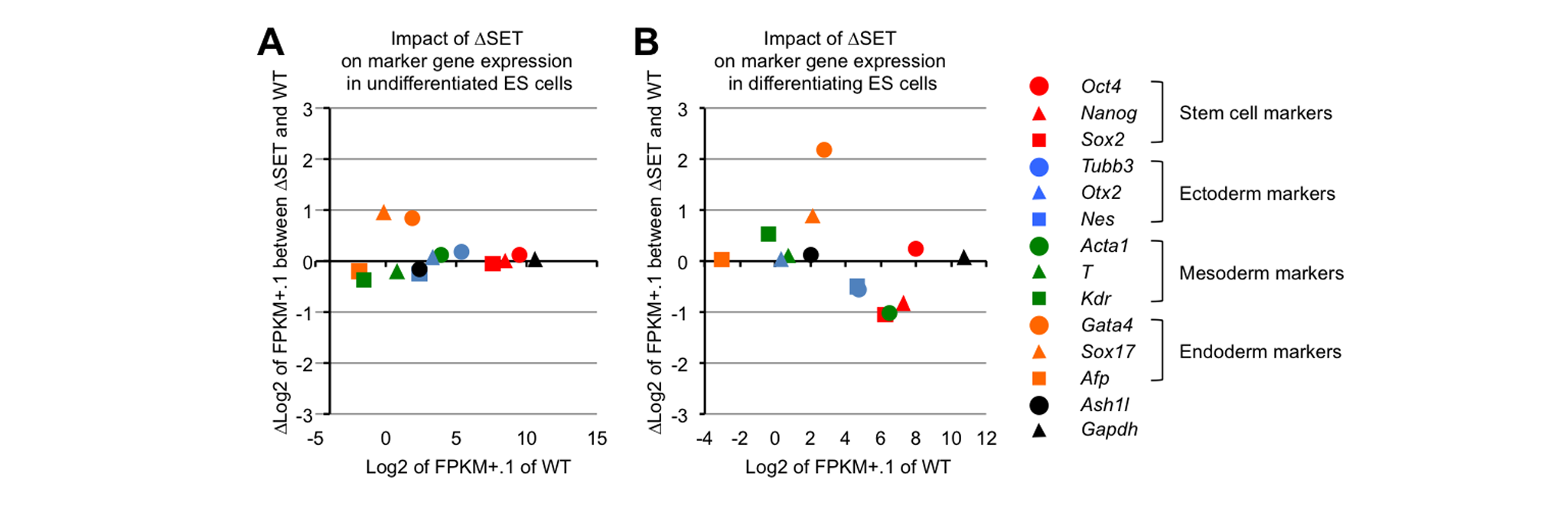

Supplement: Figure S3 — RNA-Seq data for marker gene expression. The results of indicated marker genes are plotted on the graphs using modified FPKM values. The x-axis corresponds to expression levels of each gene (shown as log2 transformation of each FPKM value plus 0.1), and the y-axis corresponds to fold change in gene expression levels between ΔSET ES cells and wild-type cells (shown as Δlog2 transformation). (A) Undifferentiated ES cells. (B) Differentiating ES cells. (TIF) [file pgen.1003897.s003.tif]

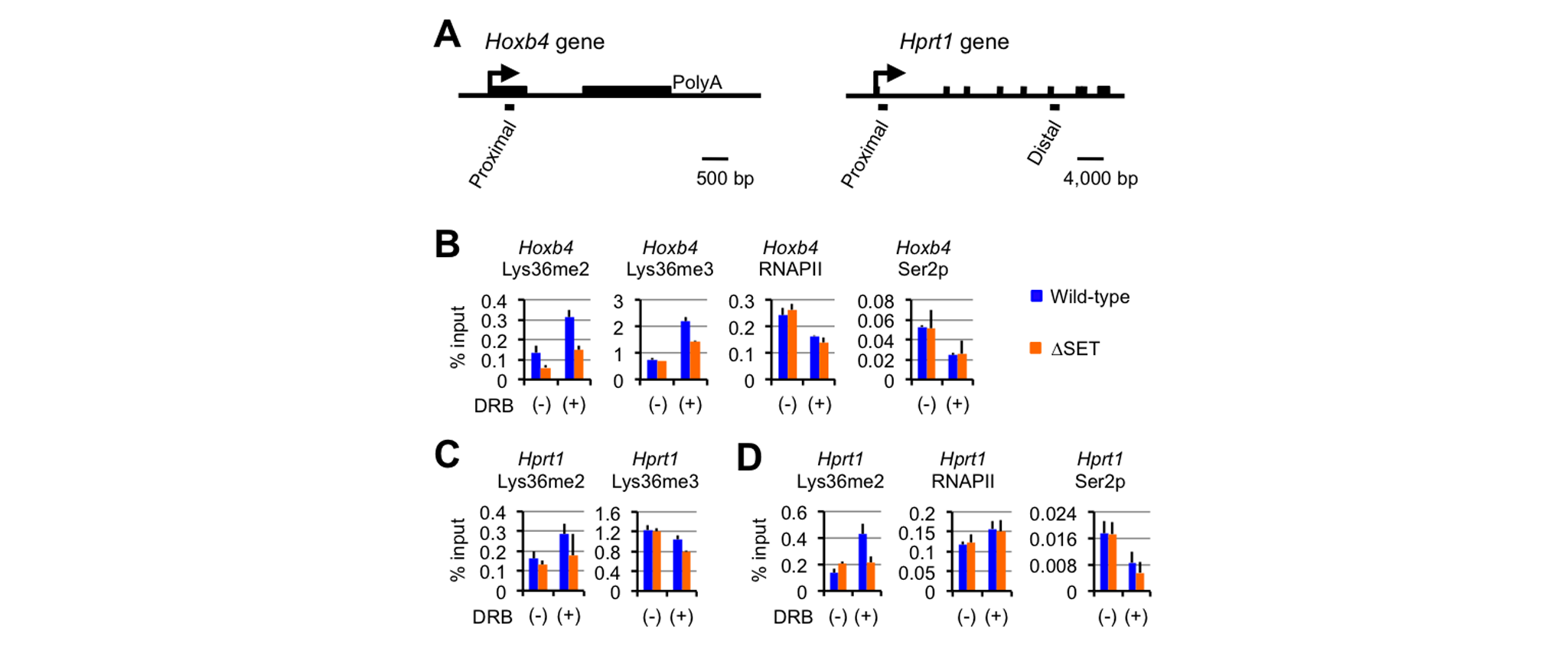

Supplement: Figure S4 — ChIP assays of histone modifications for Hoxb4 and Hprt1 in differentiating ES cells. (A) Diagrams of Hoxb4 and Hprt1 genes. Black boxes represent exons. (B–D) ChIP assays of histone modifications and the status of RNAPII in differentiating ES cells either with (+) or without (−) DRB treatment. The antibodies used are indicated at the top of each graph. The results are represented as means and s.d. (B) The promoter-proximal coding region of Hoxb4 was analyzed. (C) The promoter-proximal coding region of Hprt1 was analyzed. (D) The distal coding region of Hprt1 was analyzed. (TIF) [file pgen.1003897.s004.tif]

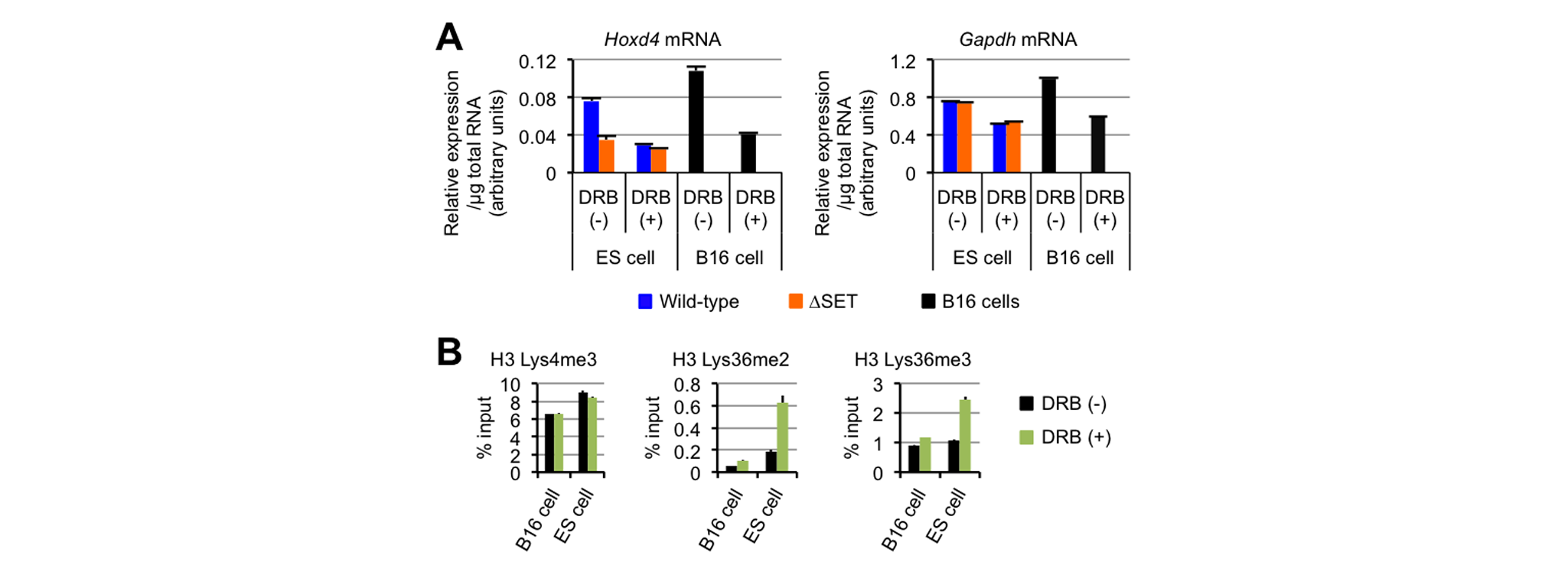

Supplement: Figure S5 — Comparison of DRB-response between ES cells and B16 cells. (A) Quantitative RT-PCR analyses of Hoxd4 and Gapdh mRNAs in differentiating ES cells and a melanoma cell line, B16. ES cells were cultured in the presence of RA (see the culture protocol shown in Figure 1D). Hoxd4 was constitutively active in B16 cells without addition of RA. The left panel depicts expression of Hoxd4 mRNA in the presence (+) or absence (−) of DRB. The right panel depicts expression of Gapdh mRNA. The results are represented as the means and s.d. of three independent PCR reactions. (B) RA-dependent increases in Lys36me2/3 levels of Hoxd4 chromatin in response to DRB. ChIP assays of B16 cells and differentiating ES cells either with (green bars) or without (black bars) DRB treatment. The promoter-proximal coding region of Hoxd4 in each cell was analyzed. The antibodies used are indicated at the top of each graph. The results are represented as means and s.d. (TIF) [file pgen.1003897.s005.tif]

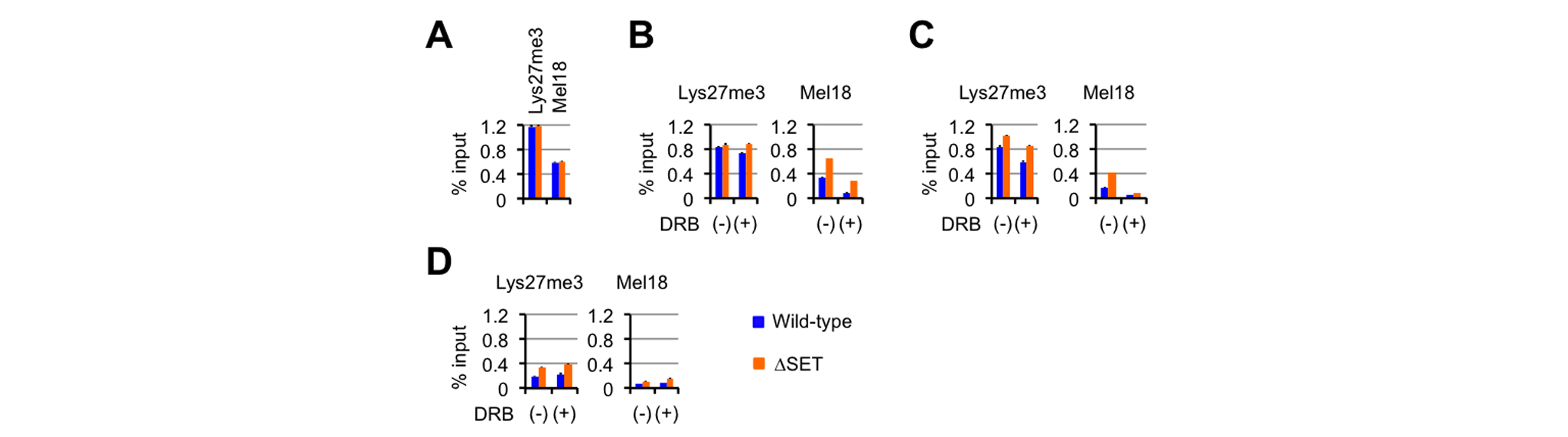

Supplement: Figure S6 — Exclusion of the PRCs occurs in a transcription-independent manner. ChIP assays of Lys27me3 and Mel18 in differentiating ES cells either with (+) or without (−) DRB treatment. (A) Occupancies of Lys27me3 and Mel18 in the promoter-proximal coding region of Hoxd4 before addition of RA. (B and C) DRB was added to the culture medium prior to RA, resulting in induction over 16 hours. The promoter-proximal (B) and distal (C) coding regions of Hoxd4 were analyzed. In (B), the same dataset as in Figure 5D was used. (D) RA was added to the culture medium prior to DRB as shown in Figure 1D, resulting in induction over 48 hours. The antibodies used are indicated at the top of each graph. The results are represented as means and s.d. (TIF) [file pgen.1003897.s006.tif]

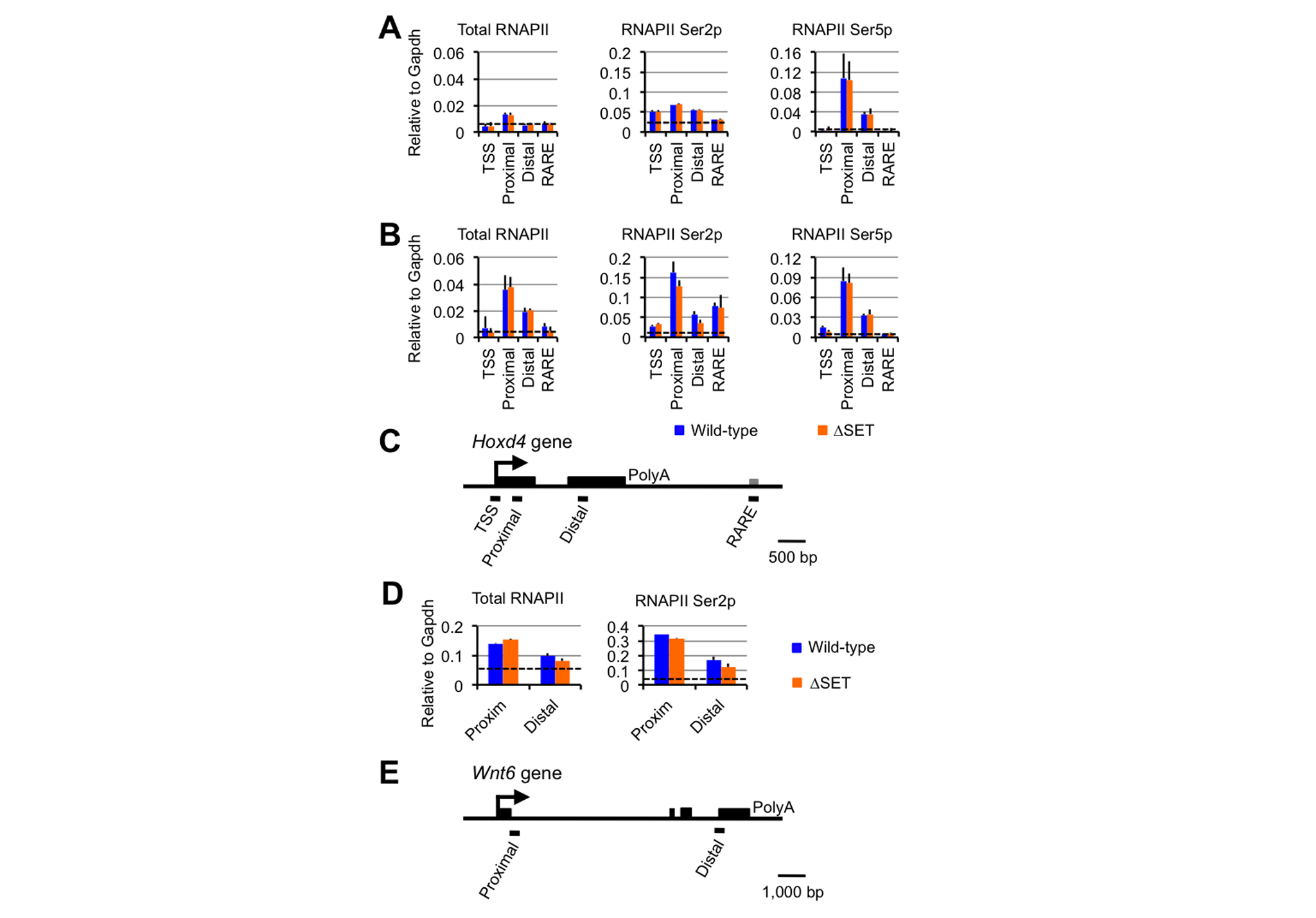

Supplement: Figure S7 — The status of RNAPII is mostly unaffected in ΔSET ES cells. (A and B) ChIP assays of various regions of Hoxd4 in differentiating ES cells before (A) or after (B) addition of RA. The results are represented as relative values that were obtained by normalizing each result to Gapdh in each cell type. Error bars represent s.d. of three independent ChIP experiments. The antibodies used are indicated above each graph. Broken lines show approximate levels of ChIP signals in the Il2ra promoter. We found that RNAPII was relatively enriched in the promoter-proximal region even before Hoxd4 activation (A), demonstrating one of the features of promoter-proximal pausing of the poised RNAPII. After RA treatment, the RNAPII levels in the coding regions were increased in both wild-type and ΔSET ES cells to a similar extent (B), suggesting that the recruitment and progression of RNAPII were not affected in ΔSET ES cells. Similar results were obtained with the phosphorylation levels of Ser2 (Ser2p) and Ser5 (Ser5p) at the carboxyl-terminal domain of RNAPII; however, the Ser2p levels in the coding regions of ΔSET ES cells were observed to be slightly affected (B). (C) A diagram of the Hoxd4 gene. Black and grey boxes represent exons and a 3′ RARE, respectively. Black bars under the diagram indicate the regions analyzed by ChIP assays. TSS: transcription start site. (D) ChIP assays of promoter-proximal and distal coding regions of Wnt6 in differentiating ES cells. The results are represented as relative values that were obtained by normalizing each result to Gapdh in each cell type. Error bars represent the s.d. of three independent ChIP experiments. The antibodies used are indicated above each graph. Broken lines show approximate levels of ChIP signals in the Il2ra promoter. (E) A diagram of the Wnt6 gene. Black boxes represent exons. Black bars under the diagram indicate the regions analyzed by ChIP assays. (TIF) [file pgen.1003897.s007.tif]

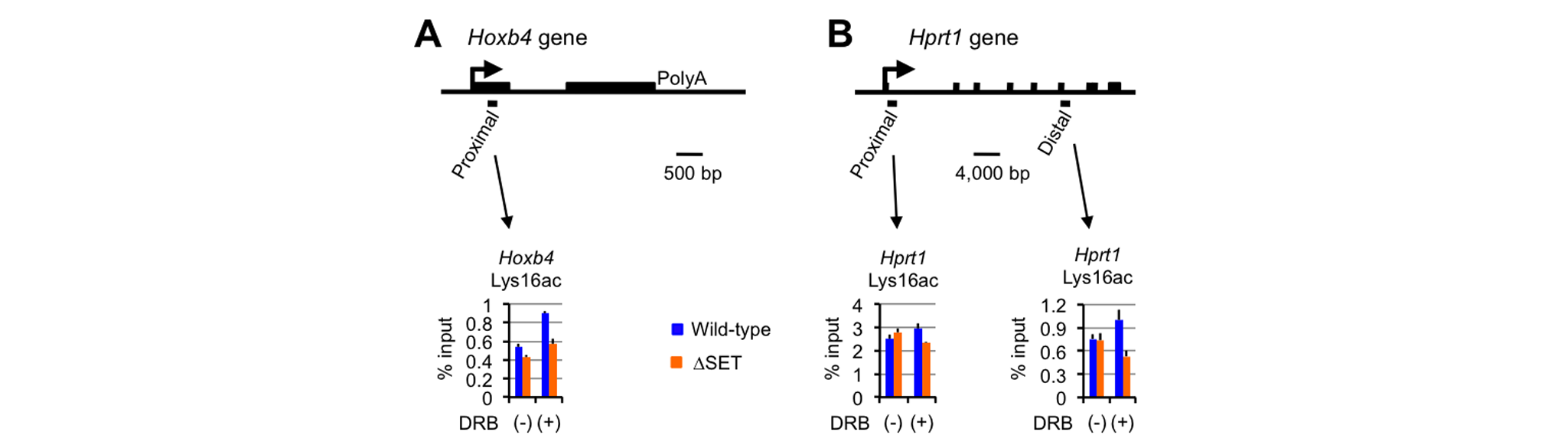

Supplement: Figure S8 — ChIP assays of histone H4 Lys16 acetylation for Hoxb4 and Hprt1 in differentiating ES cells. Diagrams of Hoxb4 and Hprt1 genes are shown on top of each ChIP result. Black boxes represent exons. ChIP assays were performed using differentiating ES cells either with (+) or without (−) DRB treatment. The results are represented as means and s.d. (A) The promoter-proximal coding region of Hoxb4 was analyzed. (B) The promoter-proximal (left) and distal (right) coding region of Hprt1 was analyzed. (TIF) [file pgen.1003897.s008.tif]

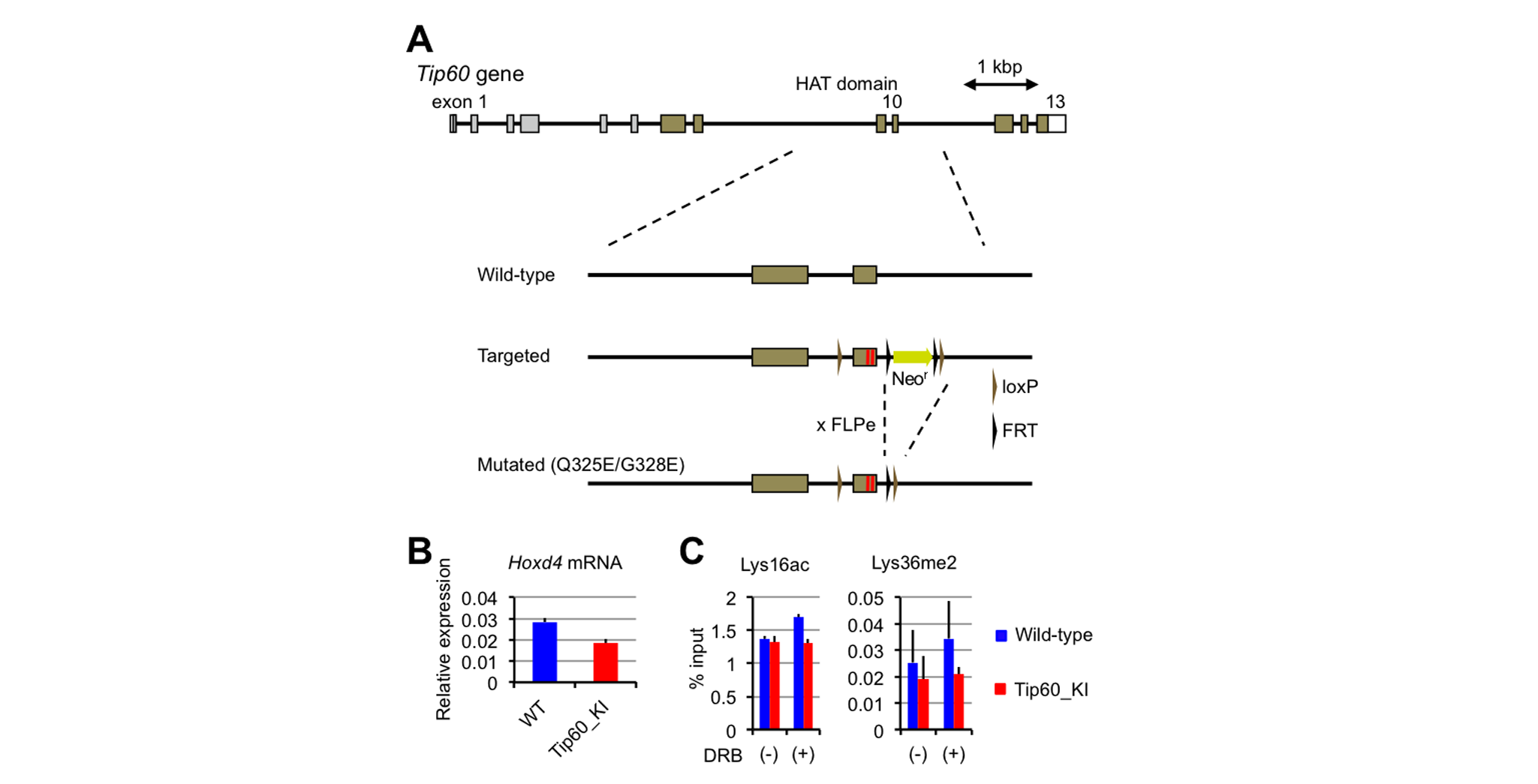

Supplement: Figure S9 — Generation of Tip60 knock-in mutant ES cells. (A) Schematic representation of the strategy used for targeted replacement of exon 10 in the Tip60 gene. The mutated exon 10 encoding a part of the histone acetyltransferase domain with its flanking introns was floxed by loxP sequences. FLPe-mediated recombination resulted in the generation of the mutated allele (Q325E and G328E, heterozygote). Red bars represent mutations in exon 10. (B) RT-PCR analysis of RA-induced Hoxd4 mRNA expression. (C) ChIP assays of histone modifications in differentiating ES cells either with (+) or without (−) DRB treatment. The promoter-proximal coding region of Hoxd4 was analyzed. The antibodies used are indicated at the top of each graph. The results are represented as means and s.d. Likely due to the heterozygosity of the knock-in mutation, we observed a mild difference in the levels of Lys16ac between wild-type and knock-in mutant ES cells. (TIF) [file pgen.1003897.s009.tif]

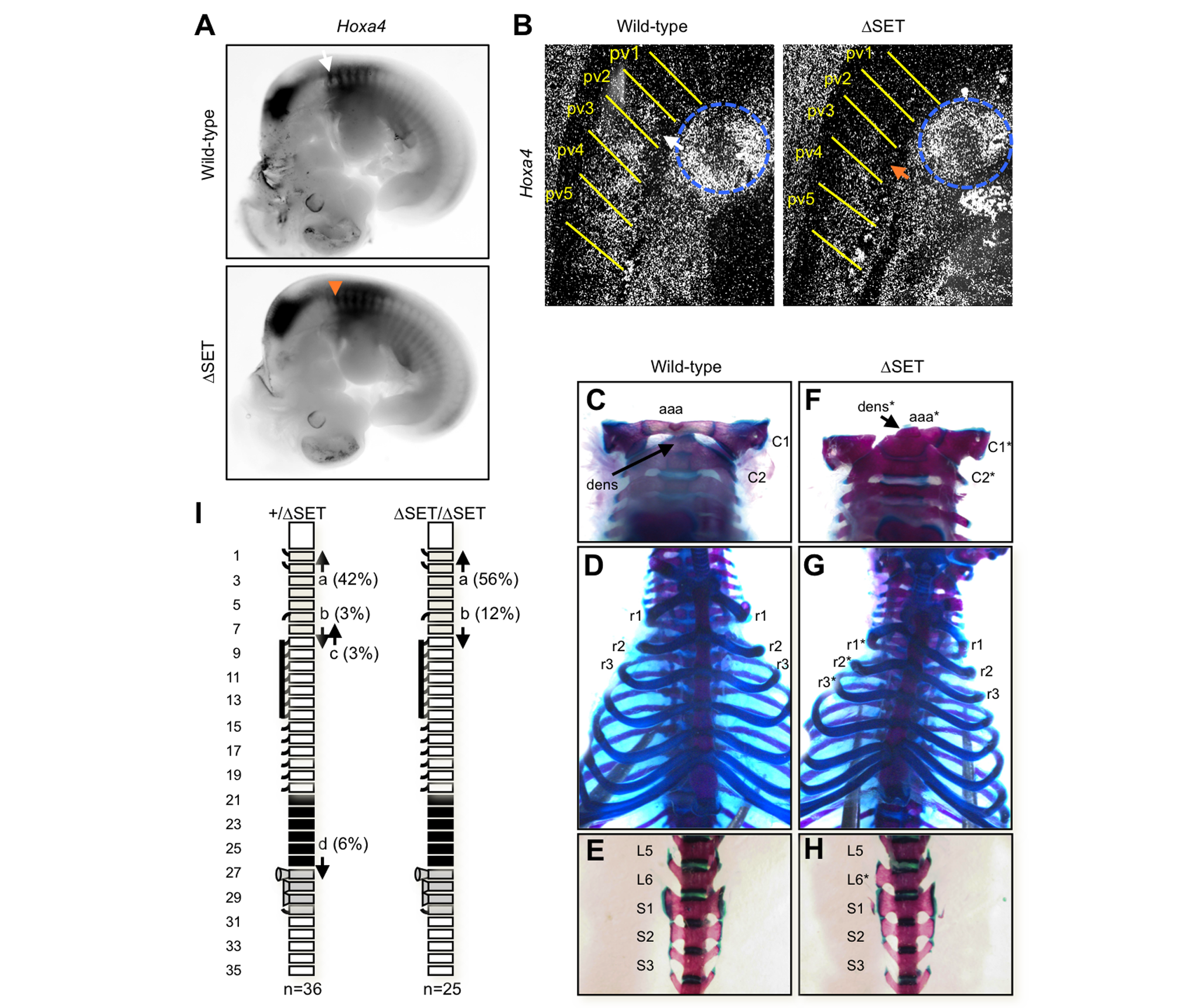

Supplement: Figure S10 — In vivo analyses of Ash1l ΔSET mutant mice. (A and B) In situ hybridization analysis of Hoxa4 mRNA in E11.5-embryos. In (A), results of whole-mount in situ hybridizations for Hoxa4 mRNA are shown. Normal (white arrows) and affected (orange arrowheads) anterior expression boundaries at the paraxial mesoderm in wild-type and ΔSET embryos. In (B), results of in situ hybridizations for Hoxa4 mRNA are shown in a representative cross-sectional image. A radio-isotope-labeled antisense-riboprobe was used for the detection of the mRNA. Yellow lines represent boundaries between each pre-vertebra (pv). Each arrow indicates the most anterior boundaries of Hoxa4 mRNA expression. An atrial chamber of the heart in each embryo is encircled by a blue-broken line. (C–H) Typical skeletal phenotypes of Ash1l ΔSET mice. Ventral views of the axial skeleton are shown. (C, D and E) Wild-type, (F, G, and H) ΔSET mice. (C and F) The cervical region. In (F), the dens of the C2* is fused to the C1*, affecting the formation of the anterior arch of atlas (aaa*). (F and G) The thoracic region. In (G), the abnormal rib cage is shown. Identities of sternoclavicular joints are mismatched between the left and right sides (for example, r2 to r1*). (E and H) The lumbo-sacral region. In (H), the transverse process of the L6* is fused to that of the S1. (I) Schematic representation summarizing the homeotic transformations. The vertebrae are numbered serially from the C1 vertebra, in which the cervical region is from 1 to 7, the thoracic region is from 8 to 20, the lumbar region is from 21 to 26, and the sacral region is from 27 to 30. a, the C2-to-C1 transformation. b, the C7-to-T1 transformation. c, the T1-to-C7 transformation. d, the L6-to-S1 transformation. (TIF) [file pgen.1003897.s010.tif]

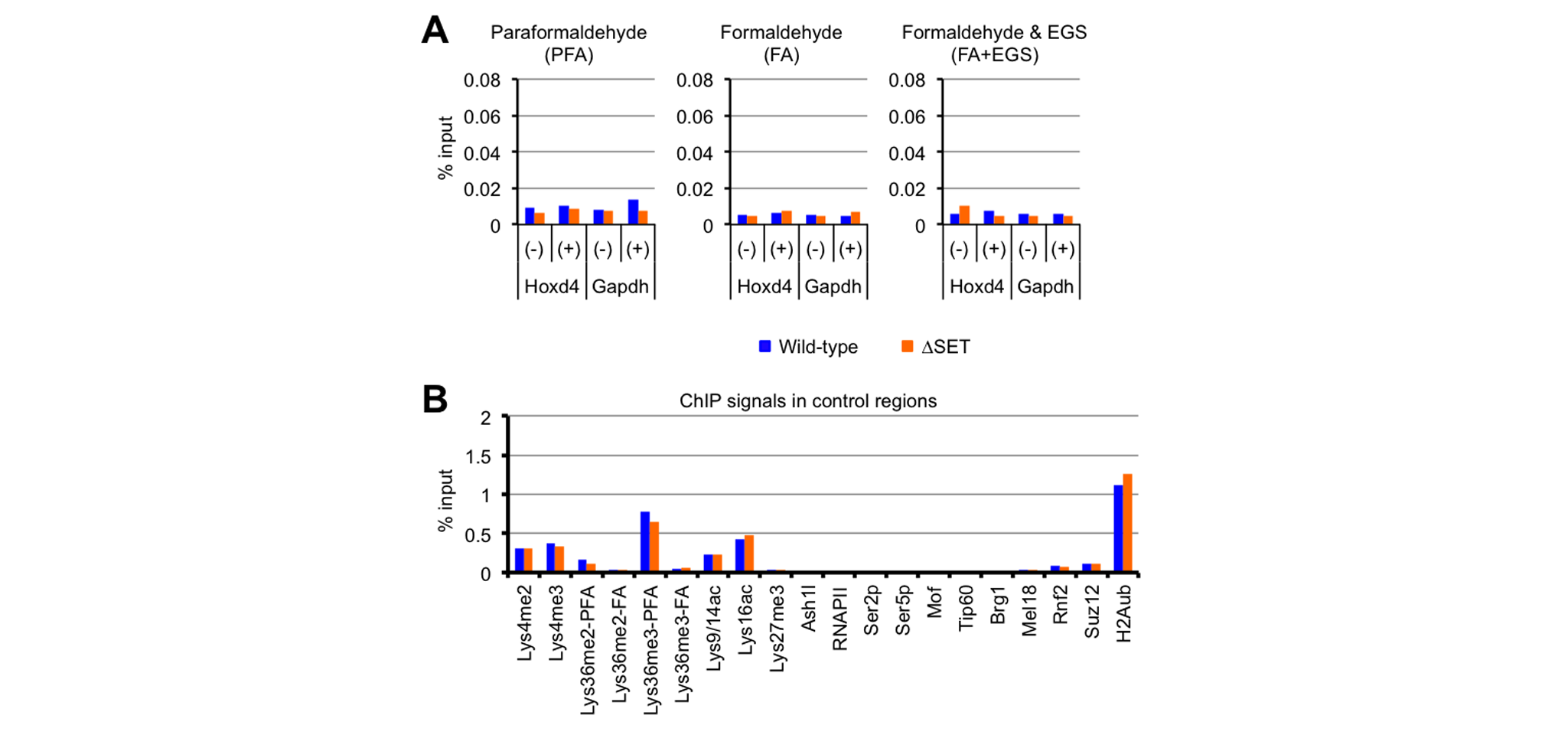

Supplement: Figure S11 — Background and control signals in ChIP assays. (A) Background ChIP signals in the promoter-proximal regions of indicated genes with (+) or without (−) DRB treatment are shown. The fixatives that were used are indicated above each graph (see Table S5 for fixation protocols). The results are represented as the means. Most background ChIP signals were around 0.01% input and are subtracted from most of the respective results. (B) Control ChIP signals in either a promoter-proximal coding region of Gapdh (for Lys27me3, Mel18, Suz12, H2Aub, and Rnf2) or a promoter region of Il2ra (for the others). ChIP assays were performed using indicated antibodies and approximate levels of each result are indicated in relevant figures. (TIF) [file pgen.1003897.s011.tif]
