# Supplementary material for: Ash1l Methylates Lys36 of Histone H3 Independently of Transcriptional Elongation to Counteract Polycomb Silencing
Source: PLoS Genet. 2013 Nov 7;9(11):e1003897. doi: 10.1371/journal.pgen.1003897 (PMC3820749; doi:10.1371/journal.pgen.1003897)
Supplement: Text S1 — Supplemental Materials and Methods. Remaining materials and methods are described here. (DOCX) [file pgen.1003897.s017.docx]

**Supplemental Materials and Methods**

**Generation of ES cells.** ES cells were obtained by intercrossing heterozygote ∆SET mice. Blastocysts were isolated from the uterus of super-ovulated pregnant female mice at E3.5. Single blastocysts were cultured on gelatin-coated plates in StemMedium (DS Pharma Biomedical) supplemented with 1,000 units/ml LIF (Chemicon) and 0.1 mM 2-Mercaptoethanol. Inner cell mass outgrowths were expanded and kept in culture in the same medium. Genotypes and sex were determined by PCR using the primer-pairs listed in Table S4.

**Generation of *Tip60* knock-in mutant ES cells.** Exon 10 of *Tip60* allele in M1 ES cells was replaced to the mutant exon encoding Q325E and G328E, equivalent to Q377E and G380E mutations in a previous report [1], by targeted homologous recombination (Figure S9). Details of the strategy used for the construction of the targeting vector are available upon request.

**Histone methyltransferase assay.** Experiments were performed as previously described with modifications [2,3]. Reactions was performed in 25 µl of reaction buffer (50 mM Tris-HCl [pH8.5], 2 mM MgCl_2_, 1 mM DTT, 6 µg GST-SET domain fusion proteins, 4 µg nucleosomes, 46.7 µM (60 nCi) S-[methyl-^14^C]-adenosyl-L-methionine) at 30˚C for 3 hours. Histones were separated by 15% SDS-PAGE, electro-transferred to PVDF membrane, stained with CBB and fluorography was performed using FLA-7000 image analyzer (Fuji Film).

**Northern blot.** A BamHI/HindIII cDNA fragment from the 3’ untranslated region of *Ash1l* was cloned from IMAGE clone 650817 (Invitrogen) and inserted into pBluescript. Single-stranded RNA probes labeled with digoxigenin-UTP were synthesized from the linearized plasmid template according to manufacturer’s instructions (Roche). Mouse total RNA panels were purchased from BD Biosciences. Five µg of total RNA was separated and blotted.

**Expression and purification of GST-fusion proteins.** cDNA encoding a AWS-SET-PostSET domain was cloned from IMAGE clone 650817 (Invitrogen) into the BamHI and SalI sites of the bacterial expression vector pGEX 6P-2 (GE Healthcare). Site-directed mutagenesis was performed to generate a cDNA encoding a H2213K mutant protein. The PCR primer-pairs used are listed in Table S4. GST-fusion proteins were induced in the bacterial strain BL21(DE3)pLysS by addition of 0.1 mM isopropyl β-D-thiogalactopyranoside at 18˚C overnight, and were purified using glutathione-sepharose beads according to manufacturer’s instruction (GE Healthcare). Eluates were analyzed by CBB staining. The eluates containing the recombinant proteins were pooled and dialyzed in buffer containing 10 mM Tris-HCl [pH7.9], 0.2 mM EDTA, 100 mM NaCl, 10% glycerol, 1mM DTT and 0.2 mM PMSF.

**Nucleosome preparation.** Core histone octamers were purified from HeLa cells as previously described [4]. Recombinant *Xenopus* histones were expressed in bacteria and purified as previously described [5]. Plasmids encoding mutant histone H3 (Lys4 to Ala, Lys36 to Ala) were generated by site-directed mutagenesis. Recombinant nucleosomes were reconstituted as previously described [6].

1. Ikura T, Ogryzko VV, Grigoriev M, Groisman R, Wang J, et al. (2000) Involvement of the TIP60 histone acetylase complex in DNA repair and apoptosis. Cell 102: 463-473.

2. Nishioka K, Chuikov S, Sarma K, Erdjument-Bromage H, Allis CD, et al. (2002) Set9, a novel histone H3 methyltransferase that facilitates transcription by precluding histone tail modifications required for heterochromatin formation. Genes Dev 16: 479-489.

3. Nishioka K, Rice JC, Sarma K, Erdjument-Bromage H, Werner J, et al. (2002) PR-Set7 is a nucleosome-specific methyltransferase that modifies lysine 20 of histone H4 and is associated with silent chromatin. Mol Cell 9: 1201-1213.

4. Ausio J, van Holde KE (1986) Histone hyperacetylation: its effects on nucleosome conformation and stability. Biochemistry 25: 1421-1428.

5. Luger K, Mäder AW, Richmond RK, Sargent DF, Richmond TJ (1997) Crystal structure of the nucleosome core particle at 2.8 A resolution. Nature 389: 251-260.

6. Nishioka K, Reinberg D (2003) Methods and tips for the purification of human histone methyltransferases. Methods 31: 49-58.
